# Characterisation of trials where marketing purposes have been influential in study design: a descriptive study

**DOI:** 10.1186/s13063-015-1107-1

**Published:** 2016-01-21

**Authors:** Virginia Barbour, Druin Burch, Fiona Godlee, Carl Heneghan, Richard Lehman, Rafael Perera, Joseph S. Ross, Sara Schroter

**Affiliations:** School of Medicine, Griffith University, Gold Coast campus, Parklands Drive, Southport, Queensland 4222 Australia; John Radcliffe Hospital, Headley Way, Headington, Oxford, Oxfordshire OX3 9DU UK; The BMJ, BMA House, London, WC1H 9JR UK; Department of Primary Care Health Science, Centre for Evidence Based Medicine, New Radcliffe House, 2nd floor, Walton Street, Jericho, OX2 6NW UK; Department of Primary Care Health Science, New Radcliffe House, 2nd floor, Walton Street, Jericho, OX2 6NW UK; Department of Primary Health Care, University of Oxford, Primary Health Care, Rosemary Rue Building, Old Road Campus, Oxford, OX3 7LF UK; Department of Medicine, Section of General Internal Medicine, Yale University School of Medicine, New Haven, CT USA

**Keywords:** Trials, Marketing trials, Publication, General Medical Journals

## Abstract

**Background:**

Analysis of trial documentation has revealed that some industry-funded trials may be done more for marketing purposes than scientific endeavour. We aimed to define characteristics of drug trials that appear to be influenced by marketing considerations and estimate their prevalence.

**Methods:**

We examined reports of randomised controlled trials of drugs published in six general medical journals in 2011. Six investigators independently reviewed all publications, characterising them as YES/MAYBE/NO suspected marketing trials, and then met to reach consensus. Blinded researchers then extracted key trial characteristics. We used blinded cluster analysis to determine if key variables could characterise the categories of trials (YES/MAYBE/NO).

**Results:**

41/194 (21 %) trials were categorised as YES, 14 (7 %) as MAYBE, 139 (72 %) as NO. All YES and MAYBE trials were funded by the manufacturer, compared with 37 % of NO trials (*p* < 0.001). A higher proportion of YES trials had authors or contributors from the manufacturer involved in study design (83 % vs. 19 %), data analysis (85 % vs.15 %) and reporting (81 % vs. 15 %) than NO trials (*p* < 0.001). There was no significant difference between groups in the median number of participants screened (*p* = 0.49), but the median number of centres recruiting participants was higher for YES compared with NO trials (171 vs. 13, *p* < 0.001). YES trials were not more likely to use a surrogate (42 % vs. 30 %; *p* = 0.38) or composite primary outcome measure (34 % vs. 19 %; *p* = 0.14) than NO trials. YES trials were often better reported in terms of blinding, safety outcomes and adverse events than NO trials. YES trials more frequently included speculation that might encourage clinicians to use the intervention outside of the study population compared to NO trials (59 % vs.37 %, *p* = 0.03). Cluster analysis based on study characteristics did not identify a clear variable structure that accurately characterised YES/MAYBE/NO trials.

**Conclusions:**

We reached consensus that a fifth of drug trials published in the highest impact general medical journals in 2011 had features that were suggestive of being designed for marketing purposes. Each of the marketing trials appeared to have a unique combination of features reported in the journal publications.

**Electronic supplementary material:**

The online version of this article (doi:10.1186/s13063-015-1107-1) contains supplementary material, which is available to authorized users.

## Background

Randomised clinical trials (RCTs) are done to reduce uncertainty over the efficacy and safety of an intervention and should be designed in such a way that they yield as much useful and unbiased information as possible from as few participants and clinical events as is necessary. However, analysis of trial documentation has revealed that some industry-funded drug trials may be done more for marketing purposes than science [1]. Confidential internal company documents have revealed drug trials designed, or heavily influenced by, the marketing departments of pharmaceutical companies [1, 2]. These trials have often been referred to as marketing or seeding trials [3, 4].

Currently, there are few documented examples of marketing trials. Nonetheless, there are reasons why marketing trials should be of concern to patients and physicians. Notably, the true research objectives – to promote the use of a medical product – may not be clear to investigators and communicated to participants. The features that suggest a trial may be considered as marketing are, however, currently unclear. Vested interests, recruitment of investigators who are frequent prescribers of competing products; disproportionally high payments given to investigators; sponsorship by the company’s sales and marketing division; minimal requirements for data leading to poor data quality and recruitment of a large number of centres have all been suggested as features of a trial designed for marketing purposes [3, 4].

Pharmaceutical companies funding, designing and conducting drug trials have rational motives for establishing and advertising evidence of product efficacy and safety in order to increase prescribing of the drug during the window before patent expiration [1, 2]. Yet, the existence of marketing trials, and their provenance is difficult to prove in the absence of confidential internal company documents [1, 2]. Even less is known about their characteristics and how marketing considerations may intrude on trials of scientific and clinical value. Accordingly, the objective of this descriptive study was to define the characteristics of trials published in major medical journals that had features suggestive of marketing trials to inform efforts to better identify marketing considerations in trial design and reporting.

## Methods

### Phase 1: estimating the prevalence of suspected marketing-driven trials in leading general medical journals

#### Scoping of marketing trial features

In order to reach a broad consensus on the characteristics of a marketing trial, which have not previously been defined, the study team met to share 24 examples of published trials one or more of us suspected of being designed partly for purposes of marketing. Based on this discussion and features of confirmed marketing trials [1–4] six characteristics of marketing-influenced trials were proposed and agreed upon by raters: 1) a high level of involvement of the product manufacturer in study design, 2) data analysis, 3) and reporting of the study, 4) recruitment of small numbers of patients from numerous study sites for a common disease when they could have been recruited without difficulty from fewer sites, 5) misleading abstracts that do not report clinically relevant findings, and 6) conclusions that focus on secondary end-points and surrogate markers.

#### Sample

We generated a list of all trials evaluating one or more drug treatments published in 2011 in the top general medical journals, based on Impact Factor (*New England Journal of Medicine (NEJM), The Journal of the American Medical Association (JAMA), Lancet, Annals of Internal Medicine, PLOS Medicine*, and *The BMJ)*. We included human drug and vaccine RCTs (excluding single patient and single arm trials). Papers describing subgroup analysis or just trial follow up data were excluded. Trials of devices were also excluded unless the device contained a drug e.g. drug eluting stents. We also excluded all studies that explicitly stated they were phase I or dosing escalation studies, but did not exclude phase II trials (or phase I studies that did not explicitly state their phase).

#### Independent rating of trials

Six members of the study team (VB, FG, RL, DB, CH, JR) independently rated included trials based on whether they were suspected to be marketing trials (YES, NO, or MAYBE). They did not use fixed criteria, but based their decisions on the extent to which they each felt the six characteristics of marketing-influenced trials described above influenced the research reported. Raters were not blinded to the journal, as successful blinding was not achievable with a team so familiar with the differences in journal formatting styles. One author [SS] then collated the independent ratings.. Trials with ≥4 independent YES ratings were categorised as suspected marketing trials (referred to as YES trials), whilst trials with ≥ 3 independent NO ratings were classified as non-marketing trials (NO trials). Trials with <3 NO and <4 Yes ratings were considered to be possible marketing trials and a consensus meeting was convened to discuss these in more depth. The initial independent ratings were collated and presented to the consensus group and then a consensus decision to assign a YES/MAYBE/NO for each of these trials was reached through discussion. VB and FG were excluded from rating trials that were published in their own journals (PLOS Medicine and The BMJ). For these trials, five ratings were collated, not six, and trials with ≥ 4 independent NO ratings were classified as non-marketing trials (NO trials).

### Phase 2: characterisation of suspected marketing trials

In the second phase we sought to extract key variables, including basic information about the trials and independent assessments of their clinical relevance and quality to see if suspected marketing trials share characteristics that differentiate them from the other trials.

#### Data extraction

The study team developed a data extraction form (see Additional file 1) to capture variables that might categorise a trial as suspected marketing or not. Three members of the team (JR, VB, DB) and one independent data extractor piloted the form for ease of completion. A further eight independent data extractors were recruited to undertake further data extraction. They were blinded to our study objectives and the categorisation of the trials and were given instructions for gathering specific variables to encourage consistency. To assess clinical relevance and trial quality of each included study we extracted data from the McMaster PLUS (Premium LiteratUre Service) database of scientifically robust studies [5].

#### Correspondence with editors

To gather further information to characterise these studies, and the degree to which marketing interests might have influenced them, we wrote to the editors of the participating journals where we suspected trials were marketing (see Additional file 2). We asked editors if they considered that they might partly be marketing trials when they were being reviewed, and how their editorial teams approach the possibility of marketing as a driver in trials. To aid our understanding and inform our analysis we also asked for editors to share in confidence copies of the anonymised peer review comments relating to these articles.

#### Survey of authors

We emailed the corresponding author of all 194 trials to invite them to complete a survey (see Additional file 3) containing further questions about the design and conduct of the trials. We were transparent in explaining the purpose of our study and included a link to our published abstract presented at the Seventh International Congress on Peer Review and Biomedical Publication [6]. Non-responders were sent one email reminder.

#### Data analysis

Initial data was collated in an Excel spreadsheet by SS and the analysis was performed using SPSS (version 18.0). We summarised data using proportions and used Chi-square and Kruskal Wallis tests to make comparisons across the three chosen categories (YES/MAYBE/NO). As the MAYBE trials were also suspected marketing trials we ran a sensitivity analysis to see if it made a difference to the results if the MAYBE trials were combined with the YES trials versus the NO trials. For this sensitivity analysis (two categories only = YES/NO) we used Chi-square and Mann Whitney U Tests.

To determine if the key variables extracted could correctly identify the three study categories (YES/MAYBE/NO) RP carried out a cluster analysis in R (version 3.1.0). Briefly, these methods use all categorical and numerical variables to determine a measure of ‘similarity’ between studies. Studies that have exactly the same values for all variables will have highest similarity (typically = 1) and those with completely different values the lowest (typically = 0). These similarities are then used to determine how close studies and clusters of studies are to each other hence defining membership to a selected number of clusters. The ‘internal structure’ of these clusters – tight groups within clusters and large differences across clusters – can be used to determine if the data show important differences between these studies – in our case MARKETING vs. NOT-MARKETING. We used a “gower” metric to account for a mixture of continuous and categorical data and “partitioning around medoids” (PAM - similar to k-means) to create the clusters [7]. The number of clusters was set at 2, 3, 4 and 5 and silhouette graphs were used to test for internal structure with values of the average silhouette width above 0.5 required to determine that at least a reasonable structure has been identified [8]. We also used dendrograms to explore how these studies group and in particular looking for a clear separation between marketing and not-marketing trials in relation to combinations of the variables. All cluster analyses were carried out blinded to the categorisation (YES/MAYBE/NO), which were only used to define consistency with membership allocation.

#### Ethics

We did not seek ethical approval for our study. The main study did not involve humans and was mainly documentary analysis so did not require ethical approval. The only involvement of humans was the author survey. Authors did not give consent to take part; participation in the survey was considered an indication of consent. Authors were free to ignore our request to provide additional information about their studies.

## Results

**Fig. 1:**
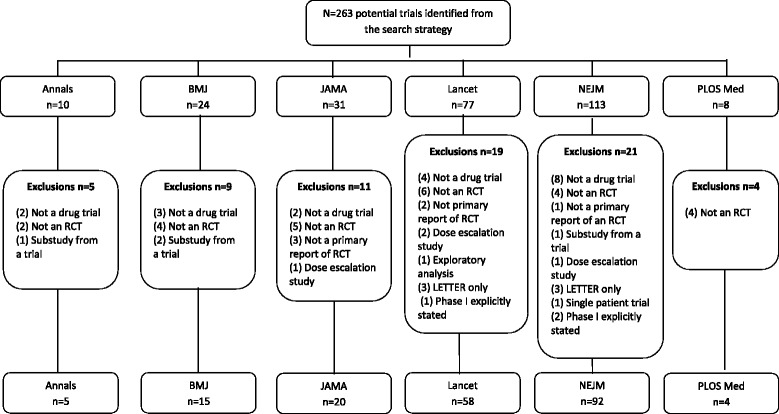
Flow chart of exclusions

Figure 1 shows that we included 194 (referenced in Additional file 4) of the 263 potential trials identified from the search strategy. Of these, 150 (77 %) were from two journals: NEJM (92) Lancet (58). Table 1 shows we categorised 41/194 (21 %) trials as YES, 14/194 (7 %) as MAYBE, and 139/194 (72 %) as NO trials. All of the trials published in The BMJ and PLOS Medicine received at least 4 or 5 NO ratings from the 5 independent raters.

**Table 1:** Number (proportion) of trials categorised as suspected marketing trials by journal following the consensus meeting

	YES	MAYBE	NO	Total number of eligible trials (%)
Annals of Internal Medicine	1	0	4	5 (3)
The BMJ	0	0	15	15 (8)
JAMA	1	1	18	20 (10)
Lancet	18	4	36	58 (30)
NEJM	21	9	62	92 (47)
PLOS Medicine	0	0	4	4 (2)
Total	41 (21)	14 (7)	139 (72)	194

**Fig. 2:**
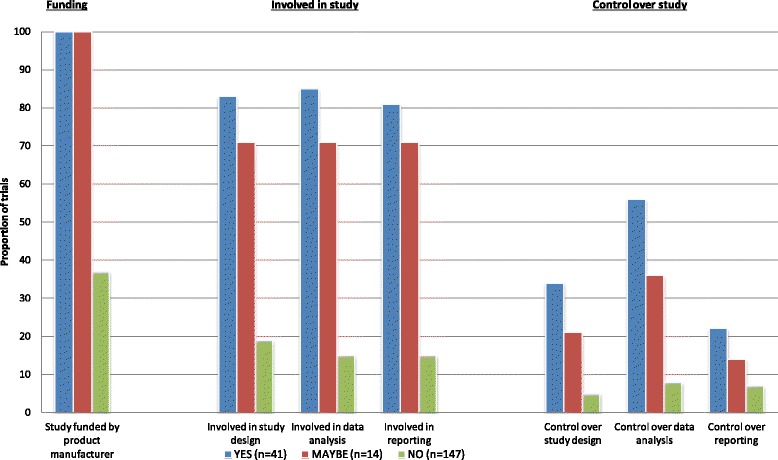
Manufacturer involvement in or control over the design, data analysis and reporting of studies

Figure 2 shows that all of the 41 YES and 14 MAYBE trials were funded by the manufacturer compared with 37 % (51/139) of the NO trials (*p* < 0.001, Table 2). YES/MAYBE trials were more likely to have authors or contributors from the manufacturer involved in the study design, data analysis and reporting of trials than NO trials (*p* < 0.001 for all three comparisons). In addition, for YES/MAYBE trials, the manufacturer was significantly (*p* < 0.001 for all three comparisons) more likely to have control of the design of the study, data analysis and reporting of the study.

**Table 2:** Authorship, writing, funding and manufacturer involvement characteristics by group

	All trials	YES trials	MAYBE trials	NO trials	*p* value (YES vs. MAYBE vs. NO)	*p* value (YES+ MAYBE vs. NO)	*p* value (MAYBE + NO vs. YES)
	*n* = 194	*n* = 41	*n* = 14	*n* = 139			
Byline author from the product manufacturer?	76 (39)	35 (85)	10 (71)	31 (22)	<.001	<.001	<.001
Median (LQ, UQ) proportion of byline authors from product manufacturer	0 (0, 16)	22 (9, 38)	19 (0, 28)	0 (0, 0)	<.001^a^	<.001^b^	<.001^b^
Byline author reporting a financial conflict of interest with the product manufacturer?^c^	126 (65)	39 (95)	14 (100)	73 (53)	<.001	<.001	<.001
Median (LQ, UQ) proportion of byline authors reporting COI with product manufacturer^c^	24 (0, 67)	82 (59, 100)	64 (49, 85)	6 (0, 40)	<.001^a^	<.001^b^	<.001^b^
Group name on the byline	111 (57)	25 (61)	9 (64)	77 (55)	.701	.415	.584
Is writing or editorial assistance in preparing the manuscript acknowledged?					.001	<.001	.002
Acknowledgements or main text	75 (39)	25 (61)	9 (64)	41 (30)			
Professional writer is a byline author	2 (1)	1 (2)	0 (0)	1 (1)			
No assistance is acknowledged	117 (60)	15 (37)	5 (36)	97 (70)			
Study explicitly funded by product manufacturer?	106 (55)	41 (100)	14 (100)	51 (37)	<.001	<.001	<.001
Manufacturer involved in the design of the study?					<.001	<.001	<.001
Yes	71 (37)	34 (83)	10 (71)	27 (19)			
No	96 (50)	4 (10)	2 (14)	90 (65)			
Not explicitly described	27 (14)	3 (7)	2 (14)	22 (16)			
Manufacturer involved in the data analysis?					<.001	<.001	<.001
Yes	66 (34)	35 (85)	10 (71)	21 (15)			
No	106 (55)	4 (10)	3 (21)	99 (71)			
Not explicitly described	22 (11)	2 (5)	1 (7)	19 (14)			
Manufacturer involved in the reporting of the study?					<.001	<.001	<.001
Yes	64 (33)	33 (81)	10 (71)	21 (15)			
No	98 (51)	3 (7)	1 (7)	94 (68)			
Not explicitly described	32 (17)	5 (12)	3 (21)	24 (17)			
Manufacturer control over the design of the study?					<.001	<.001	<.001
Yes	24 (12)	14 (34)	3 (21)	7 (5)			
No	108 (56)	9 (22)	3 (21)	96 (69)			
Not explicitly described	62 (32)	18 (44)	8 (57)	36 (26)			
Manufacturer had control over the data analysis?					<.001	<.001	<.001
Yes	39 (20)	23 (56)	5 (36)	11 (8)			
No	111 (57)	7 (17)	4 (29)	100 (72)			
Not explicitly described	44 (23)	11 (27)	5 (36)	28 (20)			
Manufacturer control over reporting of the study?					<.001	<.001	<.001
Yes	20 (10)	9 (22)	2 (14)	9 (7)			
No	111 (57)	11 (27)	4 (29)	96 (69)			
Not explicitly described	63 (33)	21 (51)	8 (57)	34 (25)			

Figures in brackets are percents unless specified otherwise.^a^ Kruskal Wallis Test.^b^ Mann Whitney *U* Test.^c^ *n* = 3 data not available as links to the COI forms do not work

YES/MAYBE trials were more likely to have at least one author from the product manufacturer on the authorship byline (*p* < 0.001) and at least one author with a declared financial conflict of interest with the product manufacturer compared with the NO trials, *p* < 0.001 (Table 2). YES/MAYBE trials were also more likely to report editorial assistance with preparing the manuscript (e.g. the use of professional writers or an indication of help with manuscript preparation in the Acknowledgements section) than NO trials, *p* = 0.001. There was no significant difference between groups in the use of group authorship on the byline (*p* = 0.70).

**Fig. 3:**
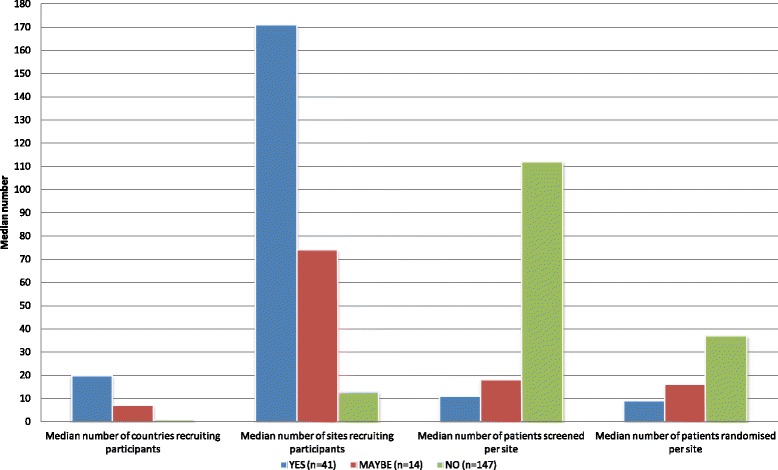
Recruitment characteristics by marketing trial category

There was no significant difference between groups in the median number of participants screened (*p* = 0.49), but the median number of centres recruiting participants was 171 for YES trials and 74 for MAYBE trials compared with 13 for NO trials (Fig. 3 and Additional file 5), *p* < 0.001. The median number of patients screened per study site was 11 in YES trials, and 18 in MAYBE trials compared with 112 in NO trials, (*p* < 0.001). The median number randomised per centre was 9, 16 and 37 (*p* < 0.001), respectively. However, the median number of months to recruit patients was significantly less for YES trials (19 months) compared to MAYBE (24 months) and NO trials (32 months), *p* = 0.003. Of note, of the 194 trials several were missing basic information about the numbers of patients screened (29 %), countries (12 %), and study sites (9 %).

A higher proportion of YES trials (32 %) reported the use of statistical imputation than NO trials (9 %) and MAYBE trials (7 %), *p* = 0.001 (see Additional file 4). YES trials were no more likely to use a surrogate primary outcome measure (*p* = 0.38) or a composite primary outcome measure (*p* = 0.14). However the Abstract’s conclusions of YES trials (39 %) were more likely to focus on surrogate outcomes (7 % MAYBE, 25 % NO; *p* = 0.05). YES trials included speculation or generalised phrasing 59 % of the time that might encourage clinicians to use the intervention outside of the study population compared to 29 % for MAYBE and 37 % for NO trials (*p* = 0.03). However, the last two results were affected if the MAYBE trials were reclassified as YES (see Additional file 4). Grouping the MAYBE trials with the YES trials and performing a two group comparison did not alter substantially the pattern of results for other outcomes.

A higher proportion of YES trials blinded clinicians (83 % vs. 43 % MAYBE and 53 % NO, *p* = 0.03), reported safety outcomes and adverse events in the Abstract (83 % vs. 54 %, *p* < 0.001) and the main text of the paper (100 % vs. 84 %, *p* = 0.006), than NO trials. There was no difference in terms of type of trial picked up in Evidence Updates (*p* = 0.36), nor in median highest clinical relevance (*p* = 0.34) and newsworthiness (*p* = 0.42) ratings.

**Fig. 4:**
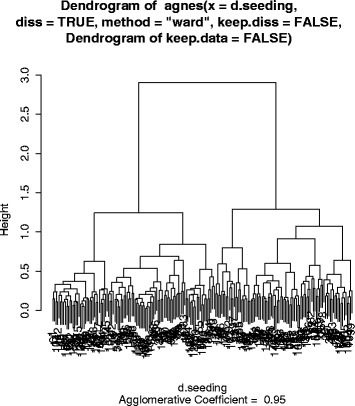
Represents how the groupings are done and are based on similarity between studies. Studies that are more ‘similar’ to each other will appear jointed earlier. The Y-axis (height) is based on the inverse of the ‘similarity’ with studies (or groups of studies) joining up at greater heights representing studies (or group of studies) that are less similar than those that join up at lower heights. The overall spread of the studies with roughly equal numbers in groups appear to show a lack of clustering structure (at least in relation to YES/NO/MAYBE) and is consistent with the silhouette graph

**Fig. 5:**
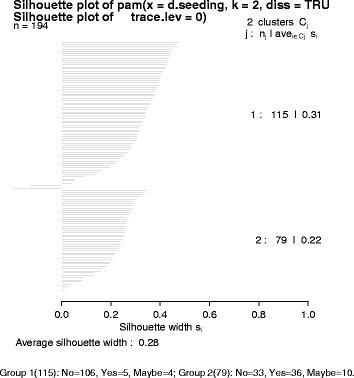
(Silhouette plot) helps with the interpretation of how many groups are needed as well as how ‘similar’ the studies are within each group. This figure shows that the two groups get a considerable number of studies instead of one group accounting for just a handful of studies. At the same time it shows that the 10.1186/s13063-015-1107-1 ‘similarity’ within groups is relatively poor with averages within group of (0.31 and 0.22). In case of adequate discrimination and grouping, these averages would be expected in the region of 0.6. The Figure presented here was typical of that found for larger values for the number of groups

In the cluster analysis we found no groupings that could identify trials as marketing versus non-marketing based on all the extracted variables (Figs. 4 and 5, Table 3). Table 3 presents how the different trials in the study were allocated assuming different number of groups (to account for possible subgroups within the YES/NO studies). Although when using only 2 or 3 groups most (36/41) of the YES trials were allocated to Group 2, this group also included a high number of NOs and MAYBEs. This is roughly equivalent to having high sensitivity (88 % of YES trials allocated there) with poor specificity. This performance did not improve by increasing the number of groups. The best average silhouette width (measure of adequate clustering) was obtained when selecting only two clusters, generating a similarity score of 0.28 suggesting that no substantial structure was identified.

**Table 3:** Cluster membership by categories

	Cluster (total)	Maybe	No	Yes
Number of Clusters = 2	1 (115)	4	106	5
	2 (79)	10	33	36
Number of Clusters = 3	1 (43)	1	42	0
	2 (78)	10	32	36
	3 (73)	3	65	5
Number of Clusters = 4	1 (43)	1	42	0
	2 (52)	9	23	20
	3 (67)	2	63	2
	4 (32)	2	11	19
Number of Clusters = 5	1 (42)	1	41	0
	2 (51)	9	23	19
	3 (34)	2	29	3
	4 (35)	0	35	0
	5 (32)	2	11	19

### Correspondence with editors and authors

We received a response from editors at all four journals where YES trials were published. Editors did not agree that marketing considerations had played a key part in any of their published studies and noted to us the underlying importance and novelty of the clinical research described by the manuscripts. For reasons of confidentiality, none of the editors were willing to share peer reviewers’ reports or any editorial notes on the decision making for these manuscripts.

From the 194 survey invitations to corresponding authors we received a delivery failure notice for 11 (6 %). Of the 183 authors 55 (30 %) completed at least some of the questionnaire. The response rate was higher for authors of MAYBE (38 %) and NO (33 %) trials compared with YES trials (18 %). Due to the low response rate we are unable to draw firm conclusions from the data.

## Discussion

Six clinical research experts and medical journal editors proposed that 21 % of drug trials published in 2011 in the leading general medical journals had characteristics consistent with the aim of marketing the product. Use of expert opinion is necessarily subjective and also open to bias. Therefore our methods aimed to reduce this bias, or at least to explore and make it explicit, and to better classify what seemed to us to be the characteristics of trials designed with marketing in mind. To achieve this we used independent data extractors to try and identify a consistent set of characteristics of marketing trials. The proposed set of marketing trials were more likely to be explicitly funded by the manufacturer, have authors or contributors from the manufacturer involved in or in control of the study design, data analysis and reporting of trials. In addition, suspected marketing trials contained speculation or generalised phrasing that might encourage clinicians to use the intervention outside of the study population.

There was an unavoidably circular aspect to our efforts, since we started with the feeling that marketing was a strong element of a number of trials and attempted to define the characteristics that contributed to that impression. Moreover, we could not define any groupings, based on the extracted variables, which could delineate trials as marketing. Furthermore, the reported methods of most of the proposed marketing trials were high quality. Our preconceptions, therefore, may have been biased by prior knowledge of marketing or seeding studies. In effect, the group of experts through consensus were able to identify a group of suspected marketing trials, but this grouping could not be validated, and remains open to experimenter’s bias: a subjective bias whereby the result is overtly influenced by the experimenters, in this case the six raters. We call upon other independent researchers to further investigate this important topic.

One characteristic, though, is worth mentioning. The proposed group of marketing trials did report recruiting large numbers of patients from multiple countries and study sites yielding a very small average number of patients per centre. Recruitment from multiple sites is more expensive and harder to manage. Legitimate reasons for recruiting from a large number of centres might include the need to get sufficient numbers of patients in a shorter period, targeted enrolment for regulatory approval purposes, and the need to ensure a diverse geographical sample. Nonetheless, compared with interventional trials conducted without industry support, it is notable that trials identified by the study team as marketing with a YES score reported on common conditions where, in the opinion of the raters, patients could easily have been recruited from a smaller number of centres in a similar time period, with less risk to data quality and at reduced cost. Involvement in the design of the study could include recruitment strategies. The justification for specific recruitment strategies are not typically reported in journal articles, protocols or trial registries so this information can only be gathered from the manufacturers or the authors of the study. We tried but failed to obtain this information from individual trial authors.

The question as to whether marketing trials exist remains controversial. At one end of the spectrum past editors such as Marcia Angell (NEJM) and Richard Smith (The BMJ) have claimed medical journals have become the advertising agencies of pharmaceutical companies [9, 10]. However, this view was not supported by the editors of the journals we contacted. To reach conclusive proof of marketing intent would require confirmatory evidence, such as access to key confidential company documentation, which we did not have.

An absence of marketing, though, may undermine implementation of beneficial interventions. As an example, tranexamic acid in trauma has been proven to be effective [11] but has not been widely used in practice; had the trial been designed with marketing in mind, recruiting smaller numbers of patients from a considerably larger number of centres, it might have been more influential (as well as more expensive). Nor is it even necessarily unethical to conduct a trial purely as a marketing exercise. The ISIS-2 trial examined the effect of streptokinase versus placebo in acute myocardial infarction [12], despite the fact that the authors were already persuaded on the basis of a meta-analysis that streptokinase worked [13]. The placebo-controlled trial of streptokinase was done not to discover a novel truth but because it was the most effective way to change medical practice. Such confirmatory trials are an important component of the evidence-base to ensure generalisability and the ability to replicate the intervention.

This study has manifest limitations. Firstly, we recognise that we are a self-selected group and other readers may not agree with our interpretation. Whilst some of us work, or have worked, for journals included in the analysis we have attempted to reduce this influence: no one individual made a determination and editors did not assess their own journal’s papers. Secondly, our team does not include clinical experts in all the diseases reported. However, three of the six raters are medical practitioners who see or have seen patients with most of the conditions reported in the included trials. Thirdly, without access to key confidential internal documents, we cannot conclusively know by whom, and with what purpose, the trials were designed. Fourthly, we only focused on RCTs whereas marketing studies may have a range of study designs and therefore the estimate proposed in this study may vary substantially. Fifthly, we only studied the large general medical journals and the problem we are trying to characterise and quantify may be more or less common in the wider universe of medical journals where most RCTs are published. Sixthly, the study team categorised the trials as YES/MAYBE/NO using just the main trial report. It is possible that they may have categorised trials differently if they assessed all the supplementary documentation accompanying the publications. Data extractors did extract data from the online supplementary files. Seventhly, contacting authors and editors did not offer confirmatory evidence of any of our findings. Finally, there was a lot of unreported information (for example the proportion of the sample who dropped out) and this may have influenced the cluster analysis. We took a pragmatic approach of sampling all trials published in six general medical journals in a particular year and it is possible that the study did not have sufficient statistical power.

This study revealed the difficulties of trying to apply a quantitative approach to characterising something as nebulous as a marketing trial. Inevitably our conclusions are more suited to start a debate than to settle it. The main issue raised is whether the current system of designing and publishing interventional trials does serve the best interests of patients and health practitioners. We hope that this research will trigger further debate on trials and remind editors, peer reviewers and readers of the need to closely scrutinise clinical trials for their design (including recruitment strategies and the role of the product manufacturer in the design, analysis and reporting of the study) as well as their findings.

## Conclusions

Manufacturers have to market their drugs but marketing imperatives should not unduly influence the design of the studies and the clinical question under investigation. In this study six raters reached a consensus that a fifth of drug trials published in the top six general medical journals were suggestive of trials designed to a large degree for marketing purposes. We were unable to create a taxonomy for marketing-influenced trials using statistical methods suggesting that individual trials have a unique combination of features reported in the journal publications. The pattern of features makes marketing-influenced studies difficult to identify by the average reader.

Further guidance is warranted to alert funders, ethics review boards, editors, peer reviewers and readers to warning signs of marketing-influenced trials. Marketing trials arise because manufacturers continue to have such a dominant role in the design, conduct and reporting of human testing of their own products. We support the idea that the design, analysis and reporting of clinical trials should only be done by truly independent investigators.

## Additional files

Additional file 1:
**Data extraction form.** (DOCX 32 kb) Additional file 2:
**Letter to editors at NEJM, JAMA, Lancet and Annals of Internal Medicine.** (DOCX 12 kb) Additional file 3:
**Author survey.** (DOCX 29 kb) Additional file 4:
**References for the trials included in the analysis.** (DOCX 37 kb) Additional file 5:
**Characteristics of trials by group.** (DOCX 35 kb)

## Abbreviations

YES: Trial categorised as a suspected marketing trial
MAYBE: Trial categorised as a possible marketing trial
NO: Trial categorised as not a marketing trial
