# Supplementary material for: Characterisation of trials where marketing purposes have been influential in study design: a descriptive study
Source: Trials. 2016 Jan 21;17:31. doi: 10.1186/s13063-015-1107-1 (PMC4720997; doi:10.1186/s13063-015-1107-1)
Supplement: Additional file 1: — Data extraction form. (DOCX 32 kb) [file 13063_2015_1107_MOESM1_ESM.docx]

| A | PDF Number: |  |
| --- | --- | --- |
| B | Full paper reference: |  |
| C | Rater: |  |
|  | **General** | |
| D | Are any of the byline (named) authors from the product manufacturer? | Yes  No  Not sure |
| E | If Yes, what proportion of byline (named) individual authors were from the product manufacturer? | of       authors |
| F | Do any of the byline (named) individual authors report a financial conflict of interest with the product manufacturer? | Yes  No  Not sure |
| G | If Yes, what proportion of byline (named) individual authors report a financial conflict of interest with the product manufacturer? | of |
| H | Is there a group name listed on the authorship byline? | Yes  No  Not sure |
| I | Is writing or editorial assistance in preparing the manuscript acknowledged? | No assistance is acknowledged  Assistance noted in Acknowledgements section or main text  Professional writer is an author |
| J | Was the study funded by the product manufacturer (excluding provisional materials)? | Yes  No  Not sure |
| K | Was the manufacturer involved in the **design** of the study? | Yes  No  It isn’t clear |
| L | Was the manufacturer involved in the **data analysis**? | Yes  No  It isn’t clear |
| M | Was the manufacturer involved in the **reporting** of the study? | Yes  No  It isn’t clear |
| N | Did the manufacturer have control over the **design** of the study? | Yes  No  It isn’t clear |
| O | Did the manufacturer have control over the **data** **analysis** of the study? | Yes  No  It isn’t clear |
| P | Did the manufacturer have control over the **reporting** of the study? | Yes  No  It isn’t clear |
| Q | Full conflict of interest (or competing interest) statement (pasted from paper): |  |
| R | Full funding statement (pasted from paper). Include statements under Role of the funding source/sponsor too. |  |
| S | Full statement about author contributions (pasted from paper): |  |
|  | **Condition and intervention(s)** | |
| T | Condition(s) studied: |  |
| U | Interventions (drugs) under investigation (please include the dose): |  |
|  | **Comparator** | |
| V | What was the comparator? |  |
| W | Was the comparator active or placebo? | Active  Active placebo  Inactive placebo |
|  | **Recruitment** | |
| X | Total number of participants screened? |  |
| Y | Total number of participants randomised? |  |
| Z | Number of countries recruiting participants? |  |
| AA | Number of centres recruiting participants? |  |
| AB | Average number of patients screened per centre? (Please calculate based on cells X&AA) |  |
| AC | Average number of patients randomised per centre? (Please calculate based on cells Y&AA) |  |
| AD | Number of months taken to recruit sample? |  |
|  | **Primary outcome** | |
| AE | What is the primary outcome? |  |
| AF | Is the primary outcome (see notes at end): | A clinical outcome  A surrogate outcome (i.e. laboratory measurement or physical sign)  Both a clinical outcome and a surrogate outcome (if more than one  primary outcome)  Not sure |
| AG | If a surrogate primary outcome (i.e. laboratory measurement or physical sign) was used, what was it? |  |
| AH | Is there a composite primary outcome? | Yes  No |
| AI | If Yes, what is the composite primary outcome? |  |
| AJ | Is the primary outcome an objective or subjective measure? | Objective  Subjective |
|  | **Secondary outcomes** |  |
| AK | What are the secondary outcomes? |  |
| AL | Are the secondary outcomes: | Clinical outcomes  Surrogate outcomes  Both clinical and surrogate outcomes  Not sure |
| AM | If a surrogate secondary outcome (i.e. laboratory measurement or physical sign) was used, what was it? |  |
| AN | Is there a composite secondary outcome? | Yes  No |
| AO | If Yes, what is the composite secondary outcome? |  |
|  | **Follow-up** | |
| AP | Length of follow-up for primary outcome? |  |
| AQ | What proportion of the sample was lost to follow-up? |  |
|  | **Trial details** | |
| AR | If stated in the paper, what is the trial phase? | Phase I  Phase II  Phase III  Phase IV  Not explicitly stated |
| AS | Open label? | Yes  No  It isn’t clear |
| AT | Was it a non-inferiority or superiority trial? | Non-inferiority  Superiority  Not stated |
| AU | Does this paper report only on a subgroup of a larger trial? | Yes  No  It isn’t clear |
| AV | Were the **treating clinicians** blinded to the intervention received? | Yes  No  It isn’t clear |
| AW | Were **participants** blinded to the intervention received? | Yes  No  It isn’t clear |
| AX | Were **outcome** **assessors** blinded to the intervention received? | Yes  No  It isn’t clear |
|  | **Analysis** |  |
| AY | Was statistical imputation used to account for missing outcome data? | Yes, this is explicitly stated  No, this is not explicitly stated |
| AZ | Was intention to treat or per protocol analysis done? | Intention to treat analysis  Per protocol analysis  Both intention to treat & per protocol  It isn’t clear |
| BA | Were there imbalances in the treatment groups at baseline? | Details: |
| BB | If Yes, could these imbalances have affected the outcome? | Yes  No  Not sure |
| BC | Were multiple subgroups created? | Yes  No |
|  | **Quality of reporting – ABSTRACT** | |
| BD | Are the results for the primary outcome clearly reported? If No, give details. | Yes  No  Details: |
| BE | Do the Abstract’s conclusions focus on **secondary outcomes**? If Yes, give details. | Yes  No  Details: |
| BF | Do the Abstract’s conclusions focus on **surrogate endpoints (**or **markers)**? If Yes, give details. | Yes  No  Details: |
| BG | Is there a discrepancy between the Results and the Conclusions? If Yes, give details. | Yes  No  Details: |
| BH | Are relative measures or absolute measures reported? | Relative measures only  Absolute measures only  Both |
| BI | Are safety outcomes/adverse events reported clearly? If No, give details. | Yes  No  Details: |
|  | **Quality of reporting – MAIN TEXT OF PAPER** | |
| BJ | Are the results for the primary outcome clearly reported? If No, give details. | Yes  No  Details: |
| BK | Does the Discussion/Conclusions focus on **secondary outcomes**? If Yes, give details. | Yes  No  Details: |
| BL | Does the Discussion/Conclusions focus on **surrogate endpoints (**or **markers)**? If Yes, give details. | Yes  No  Details: |
| BM | Are relative measures or absolute measures reported? | Relative measures only  Absolute measures only  Both |
| BN | Is the Number Needed to Treat (**NNT)** provided in the paper? | Yes  No, but it is calculable from the data  No, and it is not possible to calculate from the data  No, and I don't know if it can be calculated from the data |
| BO | Is the Number Needed to Harm (**NNH)** provided in the paper? | Yes  No, but it is calculable from the data  No, and it is not possible to calculate from the data  No, and I don't know if it can be calculated from the data |
| BP | Are safety outcomes/adverse events reported clearly? If No, give details. | Yes  No  Not reported |
| BQ | Is there a discrepancy between the Results and Discussion/Conclusions? If Yes, give details. | Yes  No  Details: |
| BR | Does the paper contain any speculation or generalised phrasing that might encourage clinicians to use the intervention outside the study population? If Yes, give details | Yes  No  Details: |
| BS | Anything else misleading in the paper? |  |
