# Supplementary material for: Characterisation of trials where marketing purposes have been influential in study design: a descriptive study
Source: Trials. 2016 Jan 21;17:31. doi: 10.1186/s13063-015-1107-1 (PMC4720997; doi:10.1186/s13063-015-1107-1)
Supplement: Additional file 3: — Author survey. (DOCX 29 kb) [file 13063_2015_1107_MOESM3_ESM.docx]

**Clinical trial reporting survey**

| **Study #:** | **Paper:** |
| --- | --- |

**Instructions for completion**

**Please click on a grey square to check the box and type in the grey shaded areas to enter free text. The boxes will expand to accommodate your answers; don’t worry if the boxes move onto the next page, keep typing.**

|  | **Background** | |
| --- | --- | --- |
| 1 | Was this study initiated by the investigators or the funder? | Investigator initiated  Funder initiated (government or non-profit funded trial)  Funder initiated (manufacturer funded trial)  Other (please specify): |
| 2 | After the study was conceived, who was responsible for study design (you may select more than one response)? | Study authors  Study funder  External advisory board  Other (please specify): |
| 3 | At the time of patient recruitment, had the drug under study already been approved by a drug licensing authority for another use? | Yes  No  If Yes, please give details: |
| 4 | Was there a Data Safety and Monitoring Board for the trial? | Yes  No  Other (please specify): |
| 5 | Was there an external Advisory Board for the trial? | Yes  No  Other (please specify): |
| 6 | Was a [Contract Research Organization](http://en.wikipedia.org/wiki/Contract_Research_Organization) (a CRO) used to facilitate trial conduct and recruitment? | Yes  No  Other (please specify): |
|  | **Methods for recruitment** | |
| 7 | How many countries and study sites were approached to serve as recruitment sites? | Number of countries:  Number of study sites: |
| 8 | How many countries and study sites agreed to serve as recruitment sites? | Number of countries:  Number of study sites: |
| 9 | How many countries and study sites actually recruited patients? | Number of countries:  Number of study sites: |
| 10 | The justification for the number of study sites and countries used in a study is not usually available to readers. Please indicate which of the following factors contributed to the selection of this number of sites and countries (you may select as many responses as applicable): | Efficient recruitment (to get sufficient numbers of patients  in a shorter period)  Feasible recruitment (to get a manageable number of patients)  Targeted enrolment for regulatory approval purposes  The need to ensure a diverse geographical sample  Utilisation of pre-existing relationships with study sites  Advice from a steering committee  Advice from a CRO (Contract Research Organization)  Other (please specify): |
| 11 | Recruiting from multiple sites can be expensive and it is harder to control data quality. If you recruited from multiple study sites, please indicate which of the following factors contributed to the need to use multiple sites (you may select as many responses as applicable): | Not applicable - we did not recruit patients from multiple study sites  Difficulties during recruitment that required the addition of study sites after enrolment had begun  Time pressures to recruit patients as quickly as possible  The need to increase the generalisability of the results  Encouragement from licensing authorities to include more study sites  Encouragement from the manufacturer to include more study sites  Other (please specify): |
| 12 | Do you consider the number of study sites you recruited in as a strength or weakness of your study design? | Study strength  Study weakness  Please give reasons for your answer: neither |
| 13 | Were there any changes made to the recruitment strategy from what appears in the initial study protocol? | Yes  No  If “Yes”, what changes and why? |
|  | **Clinical Investigators** | |
| 14 | Were any incentives used to encourage sites/clinical investigators to take part in the study? | Financial remuneration  The opportunity to be a co-author on the scientific publication resulting from the trial  Other (please specify): none |
|  | **Generalisability** | |
| 15 | Based on the inclusion/exclusion criteria used in the study, do you think that the patients enrolled were representative of typical patients with the condition under study? | Yes  No  Please give reasons for your response: |
|  | **Trial oversight, loss to follow-up and monitoring** |  |
| 16 | During the trial were there any problems with: |  |
|  | Trial oversight | Yes  No  Don’t know |
|  | Data integrity | Yes  No  Don’t know |
|  | Protocol violations | Yes  No  Don’t know |
|  | Loss to follow-up | Yes  No  Don’t know |
|  | The amount of missing data | Yes  No  Don’t know |
| 16a | If you stated “Yes” to any of the above, please give details: |  |
|  | **Marketing involvement** | |
| 17 | If the trial was funded by industry, do you know if there was any consultation with the marketing department in the design, analysis or reporting of the trial? | Yes  No  Don’t know  The trial was not supported by industry |
| 17a | If you answered Yes, please specify at what stages the marketing department was involved: |  |
|  | **Further comments** | |
| 18 | Any other comments? |  |
| 19 | If we have any further questions, would you mind if we contacted you again? | Yes  No  (If Yes, please provide your email address): |

**Thank you for your help with this research**
