# Supplementary material for: Characterisation of trials where marketing purposes have been influential in study design: a descriptive study
Source: Trials. 2016 Jan 21;17:31. doi: 10.1186/s13063-015-1107-1 (PMC4720997; doi:10.1186/s13063-015-1107-1)
Supplement: Additional file 4: — References for the trials included in the analysis. (DOCX 37 kb) [file 13063_2015_1107_MOESM4_ESM.docx]

**References for the trials included in the analysis**

1. Abdool Karim S, Naidoo K, Grobler A, Padayatchi N, Baxter C, Gray A, et al. Integration of antiretroviral therapy with tuberculosis treatment. The New England journal of medicine.365(16):1492-501.

2. Aberle D, Adams A, Berg C, Black W, Clapp J, Fagerstrom R, et al. Reduced lung-cancer mortality with low-dose computed tomographic screening. The New England journal of medicine.365(5):395-409.

3. Agnandji S, Lell B, Soulanoudjingar S, Fernandes J, Abossolo B, Conzelmann C, et al. First results of phase 3 trial of RTS,S/AS01 malaria vaccine in African children. The New England journal of medicine.365(20):1863-75.

4. Albert R, Connett J, Bailey W, Casaburi R, Cooper JAD, Criner G, et al. Azithromycin for prevention of exacerbations of COPD. The New England journal of medicine.365(8):689-98.

5. Alexander J, Lopes R, James S, Kilaru R, He Y, Mohan P, et al. Apixaban with antiplatelet therapy after acute coronary syndrome. The New England journal of medicine.365(8):699-708.

6. Andrews PJD, Avenell A, Noble DW, Campbell MK, Croal BL, Simpson WG, et al. Randomised trial of glutamine, selenium, or both, to supplement parenteral nutrition for critically ill patients. BMJ. 2011-03-17 00:00:00;342.

7. Atmar R, Bernstein D, Harro C, Al Ibrahim M, Chen W, Ferreira J, et al. Norovirus vaccine against experimental human Norwalk Virus illness. The New England journal of medicine.365(23):2178-87.

8. Bacon B, Gordon S, Lawitz E, Marcellin P, Vierling J, Zeuzem S, et al. Boceprevir for previously treated chronic HCV genotype 1 infection. The New England journal of medicine.364(13):1207-17.

9. Baigent C, Landray M, Reith C, Emberson J, Wheeler D, Tomson C, et al. The effects of lowering LDL cholesterol with simvastatin plus ezetimibe in patients with chronic kidney disease (Study of Heart and Renal Protection): a randomised placebo-controlled trial. Lancet.377(9784):2181-92.

10. Banerjee S, Hellier J, Dewey M, Romeo R, Ballard C, Baldwin R, et al. Sertraline or mirtazapine for depression in dementia (HTA-SADD): a randomised, multicentre, double-blind, placebo-controlled trial. Lancet.378(9789):403-11.

11. Bari A, Sadruddin S, Khan A, Khan IuH, Lehri I, Macleod W, et al. Community case management of severe pneumonia with oral amoxicillin in children aged 2-59 months in Haripur district, Pakistan: a cluster randomised trial. Lancet.378(9805):1796-803.

12. Barrett PN, Berezuk G, Fritsch S, Aichinger G, Hart M, El Amin W, et al. Efficacy, safety, and immunogenicity of a Vero-cell-culture-derived trivalent influenza vaccine: a multicentre, double-blind, randomised, placebo-controlled trial. Lancet.377(9767):751-9.

13. Bawaskar HS, Bawaskar PH. Efficacy and safety of scorpion antivenom plus prazosin compared with prazosin alone for venomous scorpion (Mesobuthus tamulus) sting: randomised open label clinical trial. BMJ. 2011-01-05 00:00:00;342.

14. Belch J, Hiatt W, Baumgartner I, Driver IV, Nikol S, Norgren L, et al. Effect of fibroblast growth factor NV1FGF on amputation and death: a randomised placebo-controlled trial of gene therapy in critical limb ischaemia. Lancet.377(9781):1929-37.

15. Blanc F-X, Sok T, Laureillard D, Borand L, Rekacewicz C, Nerrienet E, et al. Earlier versus later start of antiretroviral therapy in HIV-infected adults with tuberculosis. The New England journal of medicine.365(16):1471-81.

16. Boden W, Probstfield J, Anderson T, Chaitman B, Desvignes Nickens P, Koprowicz K, et al. Niacin in patients with low HDL cholesterol levels receiving intensive statin therapy. The New England journal of medicine.365(24):2255-67.

17. Bojang K, Akor F, Conteh L, Webb E, Bittaye O, Conway D, et al. Two strategies for the delivery of IPTc in an area of seasonal malaria transmission in the Gambia: a randomised controlled trial. PLoS Medicine.8(2):e1000409-e.

18. Bousser M-G, Amarenco P, Chamorro A, Fisher M, Ford I, Fox K, et al. Terutroban versus aspirin in patients with cerebral ischaemic events (PERFORM): a randomised, double-blind, parallel-group trial. Lancet.377(9782):2013-22.

19. Brocklehurst P, Farrell B, King A, Juszczak E, Darlow B, Haque K, et al. Treatment of neonatal sepsis with intravenous immune globulin. The New England journal of medicine.365(13):1201-11.

20. Brown M, McInnes G, Papst C, Zhang J, MacDonald T. Aliskiren and the calcium channel blocker amlodipine combination as an initial treatment strategy for hypertension control (ACCELERATE): a randomised, parallel-group trial. Lancet.377(9762):312-20.

21. Budde K, Becker T, Arns W, Sommerer C, Reinke P, Eisenberger U, et al. Everolimus-based, calcineurin-inhibitor-free regimen in recipients of de-novo kidney transplants: an open-label, randomised, controlled trial. Lancet.377(9768):837-47.

22. Bulbulia R, Bowman L, Wallendszus K, Parish S, Armitage J, Peto R, et al. Effects on 11-year mortality and morbidity of lowering LDL cholesterol with simvastatin for about 5 years in 20,536 high-risk individuals: a randomised controlled trial. Lancet.378(9808):2013-20.

23. Burger R, Brady M, Bookman M, Fleming G, Monk B, Huang H, et al. Incorporation of bevacizumab in the primary treatment of ovarian cancer. The New England journal of medicine.365(26):2473-83.

24. Burn J, Gerdes A-M, Macrae F, Mecklin J-P, Moeslein G, Olschwang S, et al. Long-term effect of aspirin on cancer risk in carriers of hereditary colorectal cancer: an analysis from the CAPP2 randomised controlled trial. Lancet.378(9809):2081-7.

25. Burt R, Shah S, Dill K, Grant T, Gheorghiade M, Schroeder J, et al. Autologous non-myeloablative haemopoietic stem-cell transplantation compared with pulse cyclophosphamide once per month for systemic sclerosis (ASSIST): an open-label, randomised phase 2 trial. Lancet.378(9790):498-506.

26. Busse W, Morgan W, Gergen P, Mitchell H, Gern J, Liu A, et al. Randomized trial of omalizumab (anti-IgE) for asthma in inner-city children. The New England journal of medicine.364(11):1005-15.

27. Chapman P, Hauschild A, Robert C, Haanen J, Ascierto P, Larkin J, et al. Improved survival with vemurafenib in melanoma with BRAF V600E mutation. The New England journal of medicine.364(26):2507-16.

28. Cheng G, Saleh M, Marcher C, Vasey S, Mayer B, Aivado M, et al. Eltrombopag for management of chronic immune thrombocytopenia (RAISE): a 6-month, randomised, phase 3 study. Lancet.377(9763):393-402.

29. Chimowitz M, Lynn M, Derdeyn C, Turan T, Fiorella D, Lane B, et al. Stenting versus aggressive medical therapy for intracranial arterial stenosis. The New England journal of medicine.365(11):993-1003.

30. Cockayne S, Hewitt C, Hicks K, Jayakody S, Kangâ€™ombe AR, Stamuli E, et al. Cryotherapy versus salicylic acid for the treatment of plantar warts (verrucae): a randomised controlled trial. BMJ. 2011-06-07 00:00:00;342.

31. Cohen C, Andrade Villanueva J, Clotet B, Fourie J, Johnson M, Ruxrungtham K, et al. Rilpivirine versus efavirenz with two background nucleoside or nucleotide reverse transcriptase inhibitors in treatment-naive adults infected with HIV-1 (THRIVE): a phase 3, randomised, non-inferiority trial. Lancet.378(9787):229-37.

32. Cohen D, Van Hout B, Serruys P, Mohr F, Macaya C, den Heijer P, et al. Quality of life after PCI with drug-eluting stents or coronary-artery bypass surgery. The New England journal of medicine.364(11):1016-26.

33. Cohen M, Chen Y, McCauley M, Gamble T, Hosseinipour M, Kumarasamy N, et al. Prevention of HIV-1 infection with early antiretroviral therapy. The New England journal of medicine.365(6):493-505.

34. Coleman R, Marshall H, Cameron D, Dodwell D, Burkinshaw R, Keane M, et al. Breast-cancer adjuvant therapy with zoledronic acid. The New England journal of medicine.365(15):1396-405.

35. Collaborators C-. Effect of tranexamic acid in traumatic brain injury: a nested randomised, placebo controlled trial (CRASH-2 Intracranial Bleeding Study). BMJ. 2011-07-01 00:00:00;343.

36. Connolly S, Camm AJ, Halperin J, Joyner C, Alings M, Amerena J, et al. Dronedarone in high-risk permanent atrial fibrillation. The New England journal of medicine.365(24):2268-76.

37. Connolly S, Eikelboom J, Joyner C, Diener H-C, Hart R, Golitsyn S, et al. Apixaban in patients with atrial fibrillation. The New England journal of medicine.364(9):806-17.

38. Conroy T, Desseigne F, Ychou M, Bouch O, Guimbaud R, Bcouarn Y, et al. FOLFIRINOX versus gemcitabine for metastatic pancreatic cancer. The New England journal of medicine.364(19):1817-25.

39. Cook D, Meade M, Guyatt G, Walter S, Heels Ansdell D, Warkentin T, et al. Dalteparin versus unfractionated heparin in critically ill patients. The New England journal of medicine.364(14):1305-14.

40. Corren J, Lemanske R, Hanania N, Korenblat P, Parsey M, Arron J, et al. Lebrikizumab treatment in adults with asthma. The New England journal of medicine.365(12):1088-98.

41. Cortes J, O'Shaughnessy J, Loesch D, Blum J, Vahdat L, Petrakova K, et al. Eribulin monotherapy versus treatment of physician's choice in patients with metastatic breast cancer (EMBRACE): a phase 3 open-label randomised study. Lancet.377(9769):914-23.

42. Crescenti A, Borghi G, Bignami E, Bertarelli G, Landoni G, Casiraghi GM, et al. Intraoperative use of tranexamic acid to reduce transfusion rate in patients undergoing radical retropubic prostatectomy: double blind, randomised, placebo controlled trial. BMJ. 2011-10-19 00:00:00;343.

43. de Bono J, Logothetis C, Molina A, Fizazi K, North S, Chu L, et al. Abiraterone and increased survival in metastatic prostate cancer. The New England journal of medicine.364(21):1995-2005.

44. DeFronzo R, Tripathy D, Schwenke D, Banerji M, Bray G, Buchanan T, et al. Pioglitazone for diabetes prevention in impaired glucose tolerance. The New England journal of medicine.364(12):1104-15.

45. Del Mastro L, Boni L, Michelotti A, Gamucci T, Olmeo N, Gori S, et al. Effect of the gonadotropin-releasing hormone analogue triptorelin on the occurrence of chemotherapy-induced early menopause in premenopausal women with breast cancer: a randomized trial. JAMA (Chicago, Ill).306(3):269-76.

46. Dicko A, Diallo A, Tembine I, Dicko Y, Dara N, Sidibe Y, et al. Intermittent preventive treatment of malaria provides substantial protection against malaria in children already protected by an insecticide-treated bednet in Mali: a randomised, double-blind, placebo-controlled trial. PLoS Medicine.8(2):e1000407-e.

47. Dooley M, Jayne D, Ginzler E, Isenberg D, Olsen N, Wofsy D, et al. Mycophenolate versus azathioprine as maintenance therapy for lupus nephritis. The New England journal of medicine.365(20):1886-95.

48. Fayad Z, Mani V, Woodward M, Kallend D, Abt M, Burgess T, et al. Safety and efficacy of dalcetrapib on atherosclerotic disease using novel non-invasive multimodality imaging (dal-PLAQUE): a randomised clinical trial. Lancet.378(9802):1547-59.

49. Felker GM, Lee K, Bull D, Redfield M, Stevenson L, Goldsmith S, et al. Diuretic strategies in patients with acute decompensated heart failure. The New England journal of medicine.364(9):797-805.

50. Fizazi K, Carducci M, Smith M, Damio R, Brown J, Karsh L, et al. Denosumab versus zoledronic acid for treatment of bone metastases in men with castration-resistant prostate cancer: a randomised, double-blind study. Lancet.377(9768):813-22.

51. Freeman E, Guthrie K, Caan B, Sternfeld B, Cohen L, Joffe H, et al. Efficacy of escitalopram for hot flashes in healthy menopausal women: a randomized controlled trial. JAMA (Chicago, Ill).305(3):267-74.

52. Gadde K, Allison D, Ryan D, Peterson C, Troupin B, Schwiers M, et al. Effects of low-dose, controlled-release, phentermine plus topiramate combination on weight and associated comorbidities in overweight and obese adults (CONQUER): a randomised, placebo-controlled, phase 3 trial. Lancet.377(9774):1341-52.

53. Galmiche J-P, Hatlebakk J, Attwood S, Ell C, Fiocca R, Eklund S, et al. Laparoscopic antireflux surgery vs esomeprazole treatment for chronic GERD: the LOTUS randomized clinical trial. JAMA (Chicago, Ill).305(19):1969-77.

54. Gao Smith F, Perkins G, Gates S, Young D, McAuley D, Tunnicliffe W, et al. Effect of intravenous β-2 agonist treatment on clinical outcomes in acute respiratory distress syndrome (BALTI-2): a multicentre, randomised controlled trial. Lancet.379(9812):229-35.

55. Gault EJ, Perry RJ, Cole TJ, Casey S, Paterson WF, Hindmarsh PC, et al. Effect of oxandrolone and timing of pubertal induction on final height in Turnerâ€™s syndrome: randomised, double blind, placebo controlled trial. BMJ. 2011-04-14 00:00:00;342.

56. Gerstein H, Miller M, Genuth S, Ismail Beigi F, Buse J, Goff D, et al. Long-term effects of intensive glucose lowering on cardiovascular outcomes. The New England journal of medicine.364(9):818-28.

57. Giuliano A, Palefsky J, Goldstone S, Moreira E, Penny M, Aranda C, et al. Efficacy of quadrivalent HPV vaccine against HPV Infection and disease in males. The New England journal of medicine.364(5):401-11.

58. Gladwin M, Kato G, Weiner D, Onyekwere O, Dampier C, Hsu L, et al. Nitric oxide for inhalation in the acute treatment of sickle cell pain crisis: a randomized controlled trial. JAMA (Chicago, Ill).305(9):893-902.

59. Goldhaber S, Leizorovicz A, Kakkar A, Haas S, Merli G, Knabb R, et al. Apixaban versus enoxaparin for thromboprophylaxis in medically ill patients. The New England journal of medicine.365(23):2167-77.

60. Goss P, Ingle J, Als-Martnez J, Cheung A, Chlebowski R, Wactawski Wende J, et al. Exemestane for breast-cancer prevention in postmenopausal women. The New England journal of medicine.364(25):2381-91.

61. Gpel W, Kribs A, Ziegler A, Laux R, Hoehn T, Wieg C, et al. Avoidance of mechanical ventilation by surfactant treatment of spontaneously breathing preterm infants (AMV): an open-label, randomised, controlled trial. Lancet.378(9803):1627-34.

62. Graf W, Mellgren A, Matzel K, Hull T, Johansson C, Bernstein M. Efficacy of dextranomer in stabilised hyaluronic acid for treatment of faecal incontinence: a randomised, sham-controlled trial. Lancet.377(9770):997-1003.

63. Granger C, Alexander J, McMurray JJV, Lopes R, Hylek E, Hanna M, et al. Apixaban versus warfarin in patients with atrial fibrillation. The New England journal of medicine.365(11):981-92.

64. Greenberg ER, Anderson G, Morgan D, Torres J, Chey W, Bravo L, et al. 14-day triple, 5-day concomitant, and 10-day sequential therapies for Helicobacter pylori infection in seven Latin American sites: a randomised trial. Lancet.378(9790):507-14.

65. Griffiths P, Stanton A, McCarrell E, Smith C, Osman M, Harber M, et al. Cytomegalovirus glycoprotein-B vaccine with MF59 adjuvant in transplant recipients: a phase 2 randomised placebo-controlled trial. Lancet.377(9773):1256-63.

66. Haller H, Ito S, Izzo J, Januszewicz A, Katayama S, Menne J, et al. Olmesartan for the delay or prevention of microalbuminuria in type 2 diabetes. The New England journal of medicine.364(10):907-17.

67. Hanania N, Alpan O, Hamilos D, Condemi J, Reyes Rivera I, Zhu J, et al. Omalizumab in severe allergic asthma inadequately controlled with standard therapy: a randomized trial. Annals of Internal Medicine.154(9):573-82.

68. Hanaway M, Woodle ES, Mulgaonkar S, Peddi VR, Kaufman D, First MR, et al. Alemtuzumab induction in renal transplantation. The New England journal of medicine.364(20):1909-19.

69. Havlir D, Kendall M, Ive P, Kumwenda J, Swindells S, Qasba S, et al. Timing of antiretroviral therapy for HIV-1 infection and tuberculosis. The New England journal of medicine.365(16):1482-91.

70. Hemmelgarn B, Moist L, Lok C, Tonelli M, Manns B, Holden R, et al. Prevention of dialysis catheter malfunction with recombinant tissue plasminogen activator. The New England journal of medicine.364(4):303-12.

71. Herbst R, Ansari R, Bustin F, Flynn P, Hart L, Otterson G, et al. Efficacy of bevacizumab plus erlotinib versus erlotinib alone in advanced non-small-cell lung cancer after failure of standard first-line chemotherapy (BeTa): a double-blind, placebo-controlled, phase 3 trial. Lancet.377(9780):1846-54.

72. Hoberman A, Paradise J, Rockette H, Shaikh N, Wald E, Kearney D, et al. Treatment of acute otitis media in children under 2 years of age. The New England journal of medicine.364(2):105-15.

73. Hovorka R, Kumareswaran K, Harris J, Allen JM, Elleri D, Xing D, et al. Overnight closed loop insulin delivery (artificial pancreas) in adults with type 1 diabetes: crossover randomised controlled studies. BMJ. 2011-04-14 00:00:00;342.

74. Imazio M, Brucato A, Cemin R, Ferrua S, Belli R, Maestroni S, et al. Colchicine for recurrent pericarditis (CORP): a randomized trial. Annals of Internal Medicine.155(7):409-14.

75. Iversen T, Solberg TK, Romner B, Wilsgaard T, Twisk J, Anke A, et al. Effect of caudal epidural steroid or saline injection in chronic lumbar radiculopathy: multicentre, blinded, randomised controlled trial. BMJ. 2011-09-13 00:00:00;343.

76. Jacobson I, McHutchison J, Dusheiko G, Di Bisceglie A, Reddy KR, Bzowej N, et al. Telaprevir for previously untreated chronic hepatitis C virus infection. The New England journal of medicine.364(25):2405-16.

77. Jamal S, Hamilton C, Eastell R, Cummings S. Effect of nitroglycerin ointment on bone density and strength in postmenopausal women: a randomized trial. JAMA (Chicago, Ill).305(8):800-7.

78. Jonathan E, Derrick B, Emma L, Sarah P, John D, Jane A, et al. C-reactive protein concentration and the vascular benefits of statin therapy: an analysis of 20,536 patients in the Heart Protection Study. Lancet.377(9764):469-76.

79. Jones C, Hunt D, McGowan D, Amin M, Chetner M, Bruner D, et al. Radiotherapy and short-term androgen deprivation for localized prostate cancer. The New England journal of medicine.365(2):107-18.

80. Jozwiak M, Oude Rengerink K, Benthem M, van Beek E, Dijksterhuis MGK, de Graaf I, et al. Foley catheter versus vaginal prostaglandin E2 gel for induction of labour at term (PROBAAT trial): an open-label, randomised controlled trial. Lancet.378(9809):2095-103.

81. Kakkar A, Cimminiello C, Goldhaber S, Parakh R, Wang C, Bergmann J-F. Low-molecular-weight heparin and mortality in acutely ill medical patients. The New England journal of medicine.365(26):2463-72.

82. Kappos L, Li D, Calabresi P, O'Connor P, Bar Or A, Barkhof F, et al. Ocrelizumab in relapsing-remitting multiple sclerosis: a phase 2, randomised, placebo-controlled, multicentre trial. Lancet.378(9805):1779-87.

83. Kastrati A, Neumann F-J, Schulz S, Massberg S, Byrne R, Ferenc M, et al. Abciximab and heparin versus bivalirudin for non-ST-elevation myocardial infarction. The New England journal of medicine.365(21):1980-9.

84. Kimberlin D, Whitley R, Wan W, Powell D, Storch G, Ahmed A, et al. Oral acyclovir suppression and neurodevelopment after neonatal herpes. The New England journal of medicine.365(14):1284-92.

85. Klein E, Thompson I, Tangen C, Crowley J, Lucia MS, Goodman P, et al. Vitamin E and the risk of prostate cancer: the Selenium and Vitamin E Cancer Prevention Trial (SELECT). JAMA (Chicago, Ill).306(14):1549-56.

86. Konat A, Yaro J, Oudraogo A, Diarra A, Gansan A, Soulama I, et al. Intermittent preventive treatment of malaria provides substantial protection against malaria in children already protected by an insecticide-treated bednet in Burkina Faso: a randomised, double-blind, placebo-controlled trial. PLoS Medicine.8(2):e1000408-e.

87. Krupitsky E, Nunes E, Ling W, Illeperuma A, Gastfriend D, Silverman B. Injectable extended-release naltrexone for opioid dependence: a double-blind, placebo-controlled, multicentre randomised trial. Lancet.377(9776):1506-13.

88. Krystal J, Rosenheck R, Cramer J, Vessicchio J, Jones K, Vertrees J, et al. Adjunctive risperidone treatment for antidepressant-resistant symptoms of chronic military service-related PTSD: a randomized trial. JAMA (Chicago, Ill).306(5):493-502.

89. Kumar GT, Sachdev HS, Chellani H, Rehman AM, Singh V, Arora H, et al. Effect of weekly vitamin D supplements on mortality, morbidity, and growth of low birthweight term infants in India up to age 6 months: randomised controlled trial. BMJ. 2011-05-31 00:00:00;342.

90. Launay O, van der Vliet D, Rosenberg A, Michel M-L, Piroth L, Rey D, et al. Safety and immunogenicity of 4 intramuscular double doses and 4 intradermal low doses vs standard hepatitis B vaccine regimen in adults with HIV-1: a randomized controlled trial. JAMA (Chicago, Ill).305(14):1432-40.

91. Lavine J, Schwimmer J, Van Natta M, Molleston J, Murray K, Rosenthal P, et al. Effect of vitamin E or metformin for treatment of nonalcoholic fatty liver disease in children and adolescents: the TONIC randomized controlled trial. JAMA (Chicago, Ill).305(16):1659-68.

92. Leissinger C, Gringeri A, Antmen B, Berntorp E, Biasoli C, Carpenter S, et al. Anti-inhibitor coagulant complex prophylaxis in hemophilia with inhibitors. The New England journal of medicine.365(18):1684-92.

93. Lembo A, Schneier H, Shiff S, Kurtz C, MacDougall J, Jia X, et al. Two randomized trials of linaclotide for chronic constipation. The New England journal of medicine.365(6):527-36.

94. Lienhardt C, Cook S, Burgos M, Yorke Edwards V, Rigouts L, Anyo G, et al. Efficacy and safety of a 4-drug fixed-dose combination regimen compared with separate drugs for treatment of pulmonary tuberculosis: the Study C randomized controlled trial. JAMA (Chicago, Ill).305(14):1415-23.

95. Louie T, Miller M, Mullane K, Weiss K, Lentnek A, Golan Y, et al. Fidaxomicin versus vancomycin for Clostridium difficile infection. The New England journal of medicine.364(5):422-31.

96. Lwenberg B, Pabst T, Vellenga E, van Putten W, Schouten H, Graux C, et al. Cytarabine dose for acute myeloid leukemia. The New England journal of medicine.364(11):1027-36.

97. Madhi S, Nachman S, Violari A, Kim S, Cotton M, Bobat R, et al. Primary isoniazid prophylaxis against tuberculosis in HIV-exposed children. The New England journal of medicine.365(1):21-31.

98. Maitland K, Kiguli S, Opoka R, Engoru C, Olupot Olupot P, Akech S, et al. Mortality after fluid bolus in African children with severe infection. The New England journal of medicine.364(26):2483-95.

99. Malfertheiner P, Bazzoli F, Delchier J-C, Celiski K, Gigure M, Rivire M, et al. Helicobacter pylori eradication with a capsule containing bismuth subcitrate potassium, metronidazole, and tetracycline given with omeprazole versus clarithromycin-based triple therapy: a randomised, open-label, non-inferiority, phase 3 trial. Lancet.377(9769):905-13.

100. Marcocci C, Kahaly G, Krassas G, Bartalena L, Prummel M, Stahl M, et al. Selenium and the course of mild Graves' orbitopathy. The New England journal of medicine.364(20):1920-31.

101. Martin D, Maguire M, Ying G-s, Grunwald J, Fine S, Jaffe G. Ranibizumab and bevacizumab for neovascular age-related macular degeneration. The New England journal of medicine.364(20):1897-908.

102. Martineau A, Timms P, Bothamley G, Hanifa Y, Islam K, Claxton A, et al. High-dose vitamin D(3) during intensive-phase antimicrobial treatment of pulmonary tuberculosis: a double-blind randomised controlled trial. Lancet.377(9761):242-50.

103. Martinez F, Chinchilli V, Morgan W, Boehmer S, Lemanske R, Mauger D, et al. Use of beclomethasone dipropionate as rescue treatment for children with mild persistent asthma (TREXA): a randomised, double-blind, placebo-controlled trial. Lancet.377(9766):650-7.

104. Martinson N, Barnes G, Moulton L, Msandiwa R, Hausler H, Ram M, et al. New regimens to prevent tuberculosis in adults with HIV infection. The New England journal of medicine.365(1):11-20.

105. Maughan T, Adams R, Smith C, Meade A, Seymour M, Wilson R, et al. Addition of cetuximab to oxaliplatin-based first-line combination chemotherapy for treatment of advanced colorectal cancer: results of the randomised phase 3 MRC COIN trial. Lancet.377(9783):2103-14.

106. McCormack F, Inoue Y, Moss J, Singer L, Strange C, Nakata K, et al. Efficacy and safety of sirolimus in lymphangioleiomyomatosis. The New England journal of medicine.364(17):1595-606.

107. Mega J, Braunwald E, Wiviott S, Bassand J-P, Bhatt D, Bode C, et al. Rivaroxaban in patients with a recent acute coronary syndrome. The New England journal of medicine.366(1):9-19.

108. Mega J, Hochholzer W, Frelinger A, Kluk M, Angiolillo D, Kereiakes D, et al. Dosing clopidogrel based on CYP2C19 genotype and the effect on platelet reactivity in patients with stable cardiovascular disease. JAMA (Chicago, Ill).306(20):2221-8.

109. Mehilli J, Pache J, Abdel Wahab M, Schulz S, Byrne R, Tiroch K, et al. Drug-eluting versus bare-metal stents in saphenous vein graft lesions (ISAR-CABG): a randomised controlled superiority trial. Lancet.378(9796):1071-8.

110. Meijvis SCA, Hardeman H, Remmelts HHF, Heijligenberg R, Rijkers G, van Velzen-Blad H, et al. Dexamethasone and length of hospital stay in patients with community-acquired pneumonia: a randomised, double-blind, placebo-controlled trial. Lancet.377(9782):2023-30.

111. Mintz Hittner H, Kennedy K, Chuang A. Efficacy of intravitreal bevacizumab for stage 3+ retinopathy of prematurity. The New England journal of medicine.364(7):603-15.

112. Molina J-M, Cahn P, Grinsztejn B, Lazzarin A, Mills A, Saag M, et al. Rilpivirine versus efavirenz with tenofovir and emtricitabine in treatment-naive adults infected with HIV-1 (ECHO): a phase 3 randomised double-blind active-controlled trial. Lancet.378(9787):238-46.

113. Molyneux E, Nizami S, Saha S, Huu K, Azam M, Bhutta Z, et al. 5 versus 10 days of treatment with ceftriaxone for bacterial meningitis in children: a double-blind randomised equivalence study. Lancet.377(9780):1837-45.

114. Montalescot G, Zeymer U, Silvain J, Boulanger B, Cohen M, Goldstein P, et al. Intravenous enoxaparin or unfractionated heparin in primary percutaneous coronary intervention for ST-elevation myocardial infarction: the international randomised open-label ATOLL trial. Lancet.378(9792):693-703.

115. Najjar S, Rao S, Melloni C, Raman S, Povsic T, Melton L, et al. Intravenous erythropoietin in patients with ST-segment elevation myocardial infarction: REVEAL: a randomized controlled trial. JAMA (Chicago, Ill).305(18):1863-72.

116. Navarra S, Guzmn R, Gallacher A, Hall S, Levy R, Jimenez R, et al. Efficacy and safety of belimumab in patients with active systemic lupus erythematosus: a randomised, placebo-controlled, phase 3 trial. Lancet.377(9767):721-31.

117. Neuzil K, Canh D, Thiem V, Janmohamed A, Huong V, Tang Y, et al. Immunogenicity and reactogenicity of alternative schedules of HPV vaccine in Vietnam: a cluster randomized noninferiority trial. JAMA (Chicago, Ill).305(14):1424-31.

118. Nguyen Khac E, Thevenot T, Piquet M-A, Benferhat S, Goria O, Chatelain D, et al. Glucocorticoids plus N-acetylcysteine in severe alcoholic hepatitis. The New England journal of medicine.365(19):1781-9.

119. Nicholls S, Ballantyne C, Barter P, Chapman MJ, Erbel R, Libby P, et al. Effect of two intensive statin regimens on progression of coronary disease. The New England journal of medicine.365(22):2078-87.

120. Nicholls S, Brewer HB, Kastelein JJP, Krueger K, Wang M-D, Shao M, et al. Effects of the CETP inhibitor evacetrapib administered as monotherapy or in combination with statins on HDL and LDL cholesterol: a randomized controlled trial. JAMA (Chicago, Ill).306(19):2099-109.

121. Noble P, Albera C, Bradford W, Costabel U, Glassberg M, Kardatzke D, et al. Pirfenidone in patients with idiopathic pulmonary fibrosis (CAPACITY): two randomised trials. Lancet.377(9779):1760-9.

122. Nunez O, Weinstein B, Scheinberg P, Biancotto A, Wu C, Young N. Horse versus rabbit antithymocyte globulin in acquired aplastic anemia. The New England journal of medicine.365(5):430-8.

123. O'Connor CM, Starling RC, Hernandez AF, Armstrong PW, Dickstein K, Hasselblad V, et al. Effect of nesiritide in patients with acute decompensated heart failure. The New England journal of medicine.365(1):32-43.

124. O'Connor P, Wolinsky J, Confavreux C, Comi G, Kappos L, Olsson T, et al. Randomized trial of oral teriflunomide for relapsing multiple sclerosis. The New England journal of medicine.365(14):1293-303.

125. Orban T, Bundy B, Becker D, DiMeglio L, Gitelman S, Goland R, et al. Co-stimulation modulation with abatacept in patients with recent-onset type 1 diabetes: a randomised, double-blind, placebo-controlled trial. Lancet.378(9789):412-9.

126. O'Shaughnessy J, Osborne C, Pippen J, Yoffe M, Patt D, Rocha C, et al. Iniparib plus chemotherapy in metastatic triple-negative breast cancer. The New England journal of medicine.364(3):205-14.

127. Patel M, Mahaffey K, Garg J, Pan G, Singer D, Hacke W, et al. Rivaroxaban versus warfarin in nonvalvular atrial fibrillation. The New England journal of medicine.365(10):883-91.

128. Pathmeswaran A, Ranasinha C, Jayamanne S, Samarakoon S, Hittharage A, Kalupahana R, et al. Low-dose adrenaline, promethazine, and hydrocortisone in the prevention of acute adverse reactions to antivenom following snakebite: a randomised, double-blind, placebo-controlled trial. PLoS Medicine.8(5):e1000435-e.

129. Pavel M, Hainsworth J, Baudin E, Peeters M, Hrsch D, Winkler R, et al. Everolimus plus octreotide long-acting repeatable for the treatment of advanced neuroendocrine tumours associated with carcinoid syndrome (RADIANT-2): a randomised, placebo-controlled, phase 3 study. Lancet.378(9808):2005-12.

130. Pergola P, Raskin P, Toto R, Meyer C, Huff JW, Grossman E, et al. Bardoxolone methyl and kidney function in CKD with type 2 diabetes. The New England journal of medicine.365(4):327-36.

131. Perren T, Swart A, Pfisterer J, Ledermann J, Pujade Lauraine E, Kristensen G, et al. A phase 3 trial of bevacizumab in ovarian cancer. The New England journal of medicine.365(26):2484-96.

132. Pimentel M, Lembo A, Chey W, Zakko S, Ringel Y, Yu J, et al. Rifaximin therapy for patients with irritable bowel syndrome without constipation. The New England journal of medicine.364(1):22-32.

133. Poordad F, McCone J, Bacon B, Bruno S, Manns M, Sulkowski M, et al. Boceprevir for untreated chronic HCV genotype 1 infection. The New England journal of medicine.364(13):1195-206.

134. Porto AMF, Coutinho IC, Correia JB, Amorim MMR. Effectiveness of antenatal corticosteroids in reducing respiratory disorders in late preterm infants: randomised clinical trial. BMJ. 2011-04-12 00:00:00;342.

135. Price D, Musgrave S, Shepstone L, Hillyer E, Sims E, Gilbert RFT, et al. Leukotriene antagonists as first-line or add-on asthma-controller therapy. The New England journal of medicine.364(18):1695-707.

136. Price M, Berger P, Teirstein P, Tanguay J-F, Angiolillo D, Spriggs D, et al. Standard- vs high-dose clopidogrel based on platelet function testing after percutaneous coronary intervention: the GRAVITAS randomized trial. JAMA (Chicago, Ill).305(11):1097-105.

137. Quoix E, Zalcman G, Oster J-P, Westeel V, Pichon E, Lavol A, et al. Carboplatin and weekly paclitaxel doublet chemotherapy compared with monotherapy in elderly patients with advanced non-small-cell lung cancer: IFCT-0501 randomised, phase 3 trial. Lancet.378(9796):1079-88.

138. Rahman N, Maskell N, West A, Teoh R, Arnold A, Mackinlay C, et al. Intrapleural use of tissue plasminogen activator and DNase in pleural infection. The New England journal of medicine.365(6):518-26.

139. Ramsey B, Davies J, McElvaney NG, Tullis E, Bell S, Dřevínek P, et al. A CFTR potentiator in patients with cystic fibrosis and the G551D mutation. The New England journal of medicine.365(18):1663-72.

140. Rauch S, Halpin C, Antonelli P, Babu S, Carey J, Gantz B, et al. Oral vs intratympanic corticosteroid therapy for idiopathic sudden sensorineural hearing loss: a randomized trial. JAMA (Chicago, Ill).305(20):2071-9.

141. Rayman M, Stranges S, Griffin B, Pastor Barriuso R, Guallar E. Effect of supplementation with high-selenium yeast on plasma lipids: a randomized trial. Annals of Internal Medicine.154(10):656-65.

142. Raymond E, Dahan L, Raoul J-L, Bang Y-J, Borbath I, Lombard Bohas C, et al. Sunitinib malate for the treatment of pancreatic neuroendocrine tumors. The New England journal of medicine.364(6):501-13.

143. Rcher C, Coiffier B, Haioun C, Molina T, Ferm C, Casasnovas O, et al. Intensified chemotherapy with ACVBP plus rituximab versus standard CHOP plus rituximab for the treatment of diffuse large B-cell lymphoma (LNH03-2B): an open-label randomised phase 3 trial. Lancet.378(9806):1858-67.

144. Reich K, Langley R, Papp K, Ortonne J-P, Unnebrink K, Kaul M, et al. A 52-week trial comparing briakinumab with methotrexate in patients with psoriasis. The New England journal of medicine.365(17):1586-96.

145. Rice T, Wheeler A, Thompson BT, deBoisblanc B, Steingrub J, Rock P. Enteral omega-3 fatty acid, gamma-linolenic acid, and antioxidant supplementation in acute lung injury. JAMA (Chicago, Ill).306(14):1574-81.

146. Richeldi L, Costabel U, Selman M, Kim D, Hansell D, Nicholson A, et al. Efficacy of a tyrosine kinase inhibitor in idiopathic pulmonary fibrosis. The New England journal of medicine.365(12):1079-87.

147. Rini B, Escudier B, Tomczak P, Kaprin A, Szczylik C, Hutson T, et al. Comparative effectiveness of axitinib versus sorafenib in advanced renal cell carcinoma (AXIS): a randomised phase 3 trial. Lancet.378(9807):1931-9.

148. Robert C, Thomas L, Bondarenko I, O'Day S, M D JW, Garbe C, et al. Ipilimumab plus dacarbazine for previously untreated metastatic melanoma. The New England journal of medicine.364(26):2517-26.

149. Roquilly A, Mahe P, Seguin P, Guitton C, Floch H, Tellier A, et al. Hydrocortisone therapy for patients with multiple trauma: the randomized controlled HYPOLYTE study. JAMA (Chicago, Ill).305(12):1201-9.

150. Rosenheck R, Krystal J, Lew R, Barnett P, Fiore L, Valley D, et al. Long-acting risperidone and oral antipsychotics in unstable schizophrenia. The New England journal of medicine.364(9):842-51.

151. Ross J, Quigley C, Cao D, Feuillan P, Kowal K, Chipman J, et al. Growth hormone plus childhood low-dose estrogen in Turner's syndrome. The New England journal of medicine.364(13):1230-42.

152. Salles G, Seymour J, Offner F, Lpez-Guillermo A, Belada D, Xerri L, et al. Rituximab maintenance for 2 years in patients with high tumour burden follicular lymphoma responding to rituximab plus chemotherapy (PRIMA): a phase 3, randomised controlled trial. Lancet.377(9759):42-51.

153. Samandari T, Agizew T, Nyirenda S, Tedla Z, Sibanda T, Shang N, et al. 6-month versus 36-month isoniazid preventive treatment for tuberculosis in adults with HIV infection in Botswana: a randomised, double-blind, placebo-controlled trial. Lancet.377(9777):1588-98.

154. Sandison TG, Homsy J, Arinaitwe E, Wanzira H, Kakuru A, Bigira V, et al. Protective efficacy of co-trimoxazole prophylaxis against malaria in HIV exposed children in rural Uganda: a randomised clinical trial. BMJ. 2011-03-31 00:00:00;342.

155. Sandset E, Bath PMW, Boysen G, Jatuzis D, Krv J, Lders S, et al. The angiotensin-receptor blocker candesartan for treatment of acute stroke (SCAST): a randomised, placebo-controlled, double-blind trial. Lancet.377(9767):741-50.

156. Schulman S, Parpia S, Stewart C, Rudd Scott L, Julian J, Levine M. Warfarin dose assessment every 4 weeks versus every 12 weeks in patients with stable international normalized ratios: a randomized trial. Annals of Internal Medicine.155(10):653-9, W201.

157. Schwartzentruber D, Lawson D, Richards J, Conry R, Miller D, Treisman J, et al. gp100 peptide vaccine and interleukin-2 in patients with advanced melanoma. The New England journal of medicine.364(22):2119-27.

158. Seymour M, Thompson L, Wasan H, Middleton G, Brewster A, Shepherd S, et al. Chemotherapy options in elderly and frail patients with metastatic colorectal cancer (MRC FOCUS2): an open-label, randomised factorial trial. Lancet.377(9779):1749-59.

159. Sheehan SR, Montgomery AA, Carey M, McAuliffe FM, Eogan M, Gleeson R, et al. Oxytocin bolus versus oxytocin bolus and infusion for control of blood loss at elective caesarean section: double blind, placebo controlled, randomised trial. BMJ. 2011-08-01 00:00:00;343.

160. Sherman K, Flamm S, Afdhal N, Nelson D, Sulkowski M, Everson G, et al. Response-guided telaprevir combination treatment for hepatitis C virus infection. The New England journal of medicine.365(11):1014-24.

161. Sherry N, Hagopian W, Ludvigsson J, Jain S, Wahlen J, Ferry R, et al. Teplizumab for treatment of type 1 diabetes (Protégé study): 1-year results from a randomised, placebo-controlled trial. Lancet.378(9790):487-97.

162. Slagman MCJ, Waanders F, Hemmelder MH, Woittiez A-J, Janssen WMT, Heerspink HJL, et al. Moderate dietary sodium restriction added to angiotensin converting enzyme inhibition compared with dual blockade in lowering proteinuria and blood pressure: randomised controlled trial. BMJ. 2011-07-26 00:00:00;343.

163. Slamon D, Eiermann W, Robert N, Pienkowski T, Martin M, Press M, et al. Adjuvant trastuzumab in HER2-positive breast cancer. The New England journal of medicine.365(14):1273-83.

164. Smith M, Saad F, Coleman R, Shore N, Fizazi K, Tombal B, et al. Denosumab and bone-metastasis-free survival in men with castration-resistant prostate cancer: results of a phase 3, randomised, placebo-controlled trial. Lancet.379(9810):39-46.

165. Sow S, Okoko B, Diallo A, Viviani S, Borrow R, Carlone G, et al. Immunogenicity and safety of a meningococcal A conjugate vaccine in Africans. The New England journal of medicine.364(24):2293-304.

166. Stefanini G, Kalesan B, Serruys P, Heg D, Buszman P, Linke A, et al. Long-term clinical outcomes of biodegradable polymer biolimus-eluting stents versus durable polymer sirolimus-eluting stents in patients with coronary artery disease (LEADERS): 4 year follow-up of a randomised non-inferiority trial. Lancet.378(9807):1940-8.

167. Sterling T, Villarino ME, Borisov A, Shang N, Gordin F, Bliven Sizemore E, et al. Three months of rifapentine and isoniazid for latent tuberculosis infection. The New England journal of medicine.365(23):2155-66.

168. Sundar S, Sinha P, Rai M, Verma D, Nawin K, Alam S, et al. Comparison of short-course multidrug treatment with standard therapy for visceral leishmaniasis in India: an open-label, non-inferiority, randomised controlled trial. Lancet.377(9764):477-86.

169. Sundy J, Baraf HSB, Yood R, Edwards NL, Gutierrez Urena S, Treadwell E, et al. Efficacy and tolerability of pegloticase for the treatment of chronic gout in patients refractory to conventional treatment: two randomized controlled trials. JAMA (Chicago, Ill).306(7):711-20.

170. Thera M, Doumbo O, Coulibaly D, Laurens M, Ouattara A, Kone A, et al. A field trial to assess a blood-stage malaria vaccine. The New England journal of medicine.365(11):1004-13.

171. Thtinen P, Laine M, Huovinen P, Jalava J, Ruuskanen O, Ruohola A. A placebo-controlled trial of antimicrobial treatment for acute otitis media. The New England journal of medicine.364(2):116-26.

172. Tricoci P, Huang Z, Held C, Moliterno D, Armstrong P, Van de Werf F, et al. Thrombin-receptor antagonist vorapaxar in acute coronary syndromes. The New England journal of medicine.366(1):20-33.

173. Vadillo-Ortega F, Perichart-Perera O, Espino S, Avila-Vergara MA, Ibarra I, Ahued R, et al. Effect of supplementation during pregnancy with L-arginine and antioxidant vitamins in medical food on pre-eclampsia in high risk population: randomised controlled trial. BMJ. 2011-05-19 00:00:00;342.

174. Vaglio A, Palmisano A, Alberici F, Maggiore U, Ferretti S, Cobelli R, et al. Prednisone versus tamoxifen in patients with idiopathic retroperitoneal fibrosis: an open-label randomised controlled trial. Lancet.378(9788):338-46.

175. van de Velde CJH, Rea D, Seynaeve C, Putter H, Hasenburg A, Vannetzel J-M, et al. Adjuvant tamoxifen and exemestane in early breast cancer (TEAM): a randomised phase 3 trial. Lancet.377(9762):321-31.

176. Vesikari T, Knuf M, Wutzler P, Karvonen A, Kieninger Baum D, Schmitt H-J, et al. Oil-in-water emulsion adjuvant with influenza vaccine in young children. The New England journal of medicine.365(15):1406-16.

177. Viviani S, Zinzani P, Rambaldi A, Brusamolino E, Levis A, Bonfante V, et al. ABVD versus BEACOPP for Hodgkin's lymphoma when high-dose salvage is planned. The New England journal of medicine.365(3):203-12.

178. Vogelmeier C, Hederer B, Glaab T, Schmidt H, Rutten-van Mölken MPMH, Beeh K, et al. Tiotropium versus salmeterol for the prevention of exacerbations of COPD. The New England journal of medicine.364(12):1093-103.

179. Vons C, Barry C, Maitre S, Pautrat K, Leconte M, Costaglioli B, et al. Amoxicillin plus clavulanic acid versus appendicectomy for treatment of acute uncomplicated appendicitis: an open-label, non-inferiority, randomised controlled trial. Lancet.377(9777):1573-9.

180. Wang C, Cao B, Liu Q-Q, Zou Z-Q, Liang Z-A, Gu L, et al. Oseltamivir compared with the Chinese traditional therapy maxingshigan-yinqiaosan in the treatment of H1N1 influenza: a randomized trial. Annals of Internal Medicine.155(4):217-25.

181. Wang W, Ware R, Miller S, Iyer R, Casella J, Minniti C, et al. Hydroxycarbamide in very young children with sickle-cell anaemia: a multicentre, randomised, controlled trial (BABY HUG). Lancet.377(9778):1663-72.

182. Watson JM, Kangâ€™ombe AR, Soares MO, Chuang L-H, Worthy G, Bland JM, et al. Use of weekly, low dose, high frequency ultrasound for hard to heal venous leg ulcers: the VenUS III randomised controlled trial. BMJ. 2011-03-08 00:00:00;342.

183. Webb E, Mawa P, Ndibazza J, Kizito D, Namatovu A, Kyosiimire Lugemwa J, et al. Effect of single-dose anthelmintic treatment during pregnancy on an infant's response to immunisation and on susceptibility to infectious diseases in infancy: a randomised, double-blind, placebo-controlled trial. Lancet.377(9759):52-62.

184. Wechsler M, Kelley J, Boyd IOE, Dutile S, Marigowda G, Kirsch I, et al. Active albuterol or placebo, sham acupuncture, or no intervention in asthma. The New England journal of medicine.365(2):119-26.

185. Wedemeyer H, Yurdaydn C, Dalekos G, Erhardt A, Çakaloğlu Y, Değertekin H, et al. Peginterferon plus adefovir versus either drug alone for hepatitis delta. The New England journal of medicine.364(4):322-31.

186. West K, Christian P, Labrique A, Rashid M, Shamim A, Klemm RDW, et al. Effects of vitamin A or beta carotene supplementation on pregnancy-related mortality and infant mortality in rural Bangladesh: a cluster randomized trial. JAMA (Chicago, Ill).305(19):1986-95.

187. West R, Zatonski W, Cedzynska M, Lewandowska D, Pazik J, Aveyard P, et al. Placebo-controlled trial of cytisine for smoking cessation. The New England journal of medicine.365(13):1193-200.

188. Wherrett D, Bundy B, Becker D, DiMeglio L, Gitelman S, Goland R, et al. Antigen-based therapy with glutamic acid decarboxylase (GAD) vaccine in patients with recent-onset type 1 diabetes: a randomised double-blind trial. Lancet.378(9788):319-27.

189. Yao J, Shah M, Ito T, Bohas C, Wolin E, Van Cutsem E, et al. Everolimus for advanced pancreatic neuroendocrine tumors. The New England journal of medicine.364(6):514-23.

190. Yusuf S, Healey J, Pogue J, Chrolavicius S, Flather M, Hart R, et al. Irbesartan in patients with atrial fibrillation. The New England journal of medicine.364(10):928-38.

191. Zannad F, McMurray JJV, Krum H, van Veldhuisen D, Swedberg K, Shi H, et al. Eplerenone in patients with systolic heart failure and mild symptoms. The New England journal of medicine.364(1):11-21.

192. Zeiger R, Mauger D, Bacharier L, Guilbert T, Martinez F, Lemanske R, et al. Daily or intermittent budesonide in preschool children with recurrent wheezing. The New England journal of medicine.365(21):1990-2001.

193. Zeuzem S, Andreone P, Pol S, Lawitz E, Diago M, Roberts S, et al. Telaprevir for retreatment of HCV infection. The New England journal of medicine.364(25):2417-28.

194. Zinman B, Fulcher G, Rao P, Thomas N, Endahl L, Johansen T, et al. Insulin degludec, an ultra-long-acting basal insulin, once a day or three times a week versus insulin glargine once a day in patients with type 2 diabetes: a 16-week, randomised, open-label, phase 2 trial. Lancet.377(9769):924-31.
