# Supplementary material for: Characterisation of trials where marketing purposes have been influential in study design: a descriptive study
Source: Trials. 2016 Jan 21;17:31. doi: 10.1186/s13063-015-1107-1 (PMC4720997; doi:10.1186/s13063-015-1107-1)
Supplement: Additional file 5: — Characteristics of trials by group. (DOCX 35 kb) [file 13063_2015_1107_MOESM5_ESM.docx]

**Additional file 5: Characteristics of trials by group**

|  | **All trials**  **n=194** | **YES trials**  **n=41** | **MAYBE trials**  **n=14** | **NO trials**  **n=139** | **p**  **(YES vs MAYBE vs NO)** | **p**  **(YES+ MAYBE**  **vs NO)** | **p**  **(MAYBE+NO vs YES)** |
| --- | --- | --- | --- | --- | --- | --- | --- |
| **Trial quality and clinical relevance** |  |  |  |  |  |  |  |
| Picked up in Evidence Updates † | 152 (78) | 29 (71) | 12 (86) | 111 (80) | .361 | .418 | .182 |
| Median (LQ, UQ) highest clinical relevance rating in EvidenceUpdates | 6 (6, 7) | 7 (6, 7) | 6 (6, 7) | 6 (6, 7) | .338 * | .204 ∆ | .144 ∆ |
| Median (LQ, UQ) highest newsworthiness rating in EvidenceUpdates | 6 (6, 6) | 6 (6, 6) | 6 (5, 6) | 6 (6, 6) | .420 * | .791 ∆ | .573 ∆ |
| **Recruitment **** |  |  |  |  |  |  |  |
| Median (LQ, UQ) number of countries recruiting participants  N (%) not calculable | 1 (1, 9)  23 (12) | 20 (4, 31)  3 (7) | 7 (3, 24)  5 (36) | 1 (1, 2)  15 (11) | .000 * | .000 ∆ | .000 ∆ |
| Median (LQ, UQ) number of centres recruiting participants  N (%) not calculable | 28 (8, 97)  18 (9) | 171 (90, 342)  2 (5) | 74 (17, 175)  3 (21) | 13 (5, 44)  13 (9) | .000 * | .000 ∆ | .000 ∆ |
| Median (LQ, UQ) number of months taken to recruit sample  N (%) not calculable | 29 (15, 40)  32 (16) | 19 (12, 30)  7 (17) | 24 (16, 35)  5 (36) | 32 (19, 43)  20 (14) | .003 * | .001 ∆ | .001 ∆ |
| Median (LQ, UQ) number of patients screened  N (%) not calculable | 1103 (458, 2915)  57 (29) | 1338 (682, 3334)  12 (29) | 680 (521, 10834)  6 (43) | 1075 (435, 2984)  39 (28) | .489 | .313 ∆ | .232 ∆ |
| Median (LQ, UQ) number of patients screened per centre  N (%) not calculable | 57 (15, 256)  67 (35) | 11 (8, 15)  13 (32) | 18 (12, 23)  8 (57) | 112 (42, 538)  46 (33) | .000 * | .000 ∆ | .000 ∆ |
| Median (LQ, UQ) number of patients randomised  N (%) not calculable | 620 (243, 1881)  0 (0) | 1195 (577, 4505)  0 (0) | 523 (282, 4774)  0 (0) | 437 (205, 1585)  0 (0) | .001 | .000 ∆ | .000 ∆ |
| Median (LQ, UQ) number of patients randomised per centre  N (%) not calculable | 19 (9, 101)  18 (9) | 9 (6, 14)  2 (5) | 16 (5, 18)  3 (21) | 37 (14, 171)  17 (12) | .000 * | .000 ∆ | .000 ∆ |
| **Comparator** |  |  |  |  |  |  |  |
| Active drug  Active placebo  Inactive placebo  Both active drug and placebo  Not applicable | 82 (42)  11 (6)  87 (45)  6 (3)  8 (4) | 17 (42)  1 (2)  21 (51)  2 (5)  0 (0) | 9 (64)  0 (0)  4 (29)  1 (7)  0 (0) | 56 (40)  10 (7)  62 (45)  3 (2)  8 (6) | .281 | .135 | .391 |
| **Primary outcome** |  |  |  |  |  |  |  |
| Is the primary outcome a clinical or surrogate outcome?  Clinical outcome  Surrogate outcome  Both | 115 (59)  62 (32)  17 (9) | 23 (56)  17 (42)  1 (2) | 9 (64)  4 (29)  1 (7) | 83 (60)  41 (30)  15 (11) | .383 | .197 | .140 |
| Composite primary outcome | 44 (23) | 14 (34) | 3 (21) | 27 (19) | .140 | .085 | .048 |
|  | **All trials**  **n=194** | **YES trials**  **n=41** | **MAYBE trials**  **n=14** | **NO trials**  **n=139** | **p**  **(YES vs MAYBE vs NO)** | **p**  **(YES+ MAYBE**  **vs NO)** | **p**  **(MAYBE+NO vs YES)** |
| Is the primary outcome an objective or subjective outcome?  Objective  Subjective  Both (more than one PO) | 180 (93)  11 (6)  3 (2) | 37 (90)  4 (10)  0 (0) | 14 (100)  0 (0)  0 (0) | 129 (93)  7 (5)  3 (2) | .494 | .464 | .306 |
| **Secondary outcomes** |  |  |  |  |  |  |  |
| Are the secondary outcomes clinical or surrogate outcomes?  Clinical outcomes  Surrogate outcomes  Both  No secondary outcomes reported | 85 (44)  37 (19)  65 (34)  7 (4) | 19 (46)  7 (17)  15 (37)  0 (0) | 9 (64)  2 (14)  2 (14)  1 (7) | 57 (41)  28 (20)  48 (35)  6 (4) | .441 | .570 | .530 |
| Composite secondary outcome | 40 (21) | 14 (34) | 4 (29) | 22 (16) | .029 | .009 | .016 |
| **Followup** |  |  |  |  |  |  |  |
| Median (LQ, UQ) proportion of sample lost to follow-up  Not clear or not reported | 2.2 (0.2, 8.0) n=174  n=20 (10%) | 1.5 (0.3, 5.0) n=34  n=7 (17%) | 1.9 (0.4, 12.8) n=14  n=0 (0%) | 2.9 (0.1, 9.1) n=126  n=13 (9%) | .680 * | .570 ∆ | .391 ∆ |
| Median (LQ, UQ) proportion of sample dropped out  Not clear or not reported  Not applicable  Rater left blank | 5.5 (0.4, 18.5)  n=151  n=24 (12%)  n=6 (3%)  n=13 (7%) | 16.5 (9.8, 29.3)  n=30  8 (20%)  1 (2%)  2 (5%) | 25.7 (4.3, 42.2)  n=12  1 (7%)  1 (7%)  0 (0%) | 2.2 (0.0, 12.9)  n=109  15 (11%)  4 (3%)  11 (8%) | .000 * | .000 ∆ | .000 ∆ |
| **Trial details** |  |  |  |  |  |  |  |
| Open label trial  Yes  No  Not clear  Other | 53 (27)  120 (62)  19 (9)  2 (1) | 5 (12)  35 (85)  1 (2)  0 (0) | 5 (36)  8 (57)  1 (7)  0 (0) | 43 (31)  77 (55)  17 (12)  2 (1) | .041 | .024 | .006 |
| Superiority or non-inferiority trial?  Superiority | 173 (89) | 34 (83) | 12 (86) | 127 (91) | .283 | .118 | .147 |
| Were treating clinicians blinded to the intervention received?  Yes  No  Not clear  Other (partial blinding) | 114 (59)  54 (28)  25 (13)  1 (1) | 34 (83)  4 (10)  3 (7)  (0) | 6 (43)  6 (43)  2 (14)  0 (0) | 74 (53)  44 (32)  20 (14)  1 (1) | .033 | .095 | .005 |
|  | **All trials**  **n=194** | **YES trials**  **n=41** | **MAYBE trials**  **n=14** | **NO trials**  **n=139** | **p**  **(YES vs MAYBE vs NO)** | **p**  **(YES+ MAYBE**  **vs NO)** | **p**  **(MAYBE+NO vs YES)** |
| Were participants blinded to the intervention received?  Yes  No  Not clear  Other: (partial blinding) | 116 (59)  53 (27)  24 (12)  1 (1) | 34 (83)  4 (10)  3 (7)  0 (0) | 7 (50)  5 (36)  2 (14)  0 (0) | 75 (54)  44 (32)  19 (14)  1 (1) | .060 | .065 | .008 |
| Were outcome assessors blinded to the intervention received?  Yes  No  Not clear | 108 (56)  36 (19)  50 (26) | 27 (66)  5 (12)  9 (22) | 9 (64)  2 (14)  3 (21) | 72 (52)  29 (21)  38 (27) | .521 | .203 | .302 |
| **Analysis** |  |  |  |  |  |  |  |
| Explicit statement that statistical imputation was used to account for missing outcome data | 27 (14) | 13 (32) | 1 (7) | 13 (9) | .001 | .003 | .000 |
| Intention to treat and or per protocol analysis  Intention to treat  Per protocol analysis  Both  Not clear | 127 (66)  7 (4)  39 (20)  21 (11) | 23 (56)  1 (2)  11 (27)  6 (15) | 11 (79)  0 (0)  1 (7)  2 (14) | 93 (67)  6 (4)  27 (19)  13 (9) | .554 | .587 | .430 |
| Were there imbalances in the treatment groups at baseline?  Yes  No  Not clear | 42 (22)  144 (74)  8 (4) | 10 (24)  31 (76)  0 (0) | 2 (14)  12 (86)  0 (0) | 30 (22)  101 (73)  8 (6) | .411 | .189 | .311 |
| If imbalances, could they have affected the outcome?  Yes  No  Not sure  Not applicable | 9 (5)  5 (3)  36 (19)  144 (74) | 2 (5)  1 (2)  8 (20)  30 (73) | 0 (0)  0 (0)  2 (14)  12 (86) | 7 (5)  4 (3)  26 (19)  102 (73) | .932 | .896 | .994 |
| Multiple subgroups created | 110 (57) | 26 (63) | 11 (79) | 73 (53) | .107 | .062 | .329 |
| **Quality of reporting – abstract** |  |  |  |  |  |  |  |
| Results for primary outcome clearly reported in Abstract | 187 (96) | 39 (95) | 14 (100) | 134 (96) | .700 | .989 | .623 |
| Abstract’s conclusions focus on secondary outcomes | 24 (12) | 6 (15) | 1 (7) | 17 (12) | .760 | .924 | .620 |
| Abstract’s conclusions focus on surrogate endpoints (or markers) | 52 (27) | 16 (39) | 1 (7) | 35 (25) | .048 | .417 | .047 |
| Discrepancy between the Results and the Conclusions | 21 (11) | 5 (12) | 1 (7) | 15 (11) | .871 | .981 | .750 |
| Relative or absolute measures reported:  Only relative measures  Only absolute measures  Both | 36 (19)  34 (18)  124 (64) | 7 (17)  6 (15)  28 (68) | 2 (14)  3 (21)  9 (64) | 27 (19)  25 (18)  87 (63) | .947 | .821 | .792 |
|  | **All trials**  **n=194** | **YES trials**  **n=41** | **MAYBE trials**  **n=14** | **NO trials**  **n=139** | **p**  **(YES vs MAYBE vs NO)** | **p**  **(YES+ MAYBE**  **vs NO)** | **p**  **(MAYBE+NO vs YES)** |
| Safety outcomes/adverse events reported clearly | 122 (63) | 34 (83) | 13 (93) | 79 (54) | .000 | .000 | .003 |
| **Quality of reporting – main text of paper** |  |  |  |  |  |  |  |
| Results for primary outcome clearly reported in main text | 199 (99) | 39 (95) | 14 (100) | 138 (99) | .147 | .138 | .052 |
| Discussion/Conclusions focus on secondary outcomes | 33 (17) | 10 (24) | 1 (7) | 22 (16) | .261 | .486 | .157 |
| Discussion/Conclusions focus on surrogate endpoints (or markers) | 53 (27) | 16 (39) | 1 (7) | 36 (26) | .054 | .480 | .058 |
| Relative or absolute measures reported  Only relative measures  Only absolute measures  Both | 25 (13)  24 (12)  145 (75) | 4 (10)  4 (10)  33 (81) | 2 (14)  3 (21)  9 (64) | 19 (14)  17 (12)  103 (74) | .745 | .875 | .634 |
| Was the NNT provided?  Yes  No | 11 (6)  183 (94) | 1 (2)  40 (98) | 1 (7)  13 (93) | 9 (7)  130 (94) | .599 | .441 | .314 |
| NNH provided?  Yes  No  N/A | 0 (0)  192 (99)  2 (1) | 0 (0)  41 (100)  0 (0) | 0 (0)  14 (00)  0 (0) | 0 (0)  137 (99)  2 (1) | .670 | .371 | .462 |
| Safety outcomes/adverse events reported clearly | 171 (88) | 41 (100) | 14 (100) | 116 (84) | .006 | .001 | .008 |
| Discrepancy between the Results and the Conclusions | 23 (12) | 7 (18) | 0 (0) | 16 (12) | .212 | .806 | .221 |
| Speculation or generalised phrasing that might encourage clinicians to use the intervention outside the study population | 79 (41) | 24 (59) | 4 (29) | 51 (37) | .028 | .069 | .009 |

Note 1: Values are numbers and (percents) unless otherwise stated. Numbers may not sum to 100% due to rounding.

LQ Lower quartile

UQ Upper quartile

* Kruskal Wallis Test

∆ Mann Whitney U Test

† EvidenceUpdates is a collaboration between BMJ Group and McMaster University's Health Information Research Unit. It identifies current best evidence from research, tailored to specific health care interests, to support evidence-based clinical decisions. All citations (from over 110 premier clinical journals) are pre-rated for quality with stringent criteria by research staff, and rated for clinical relevance and interest (newsworthiness) by at least three members of a worldwide panel of practicing physicians. Scores range from 1 to 7 with higher scores indicating greater relevance and usefulness. For each trial picked up by EvidenceUpdates in this study, we recorded the highest clinical relevance and newsworthiness rating given.

†† n=3 data not available as links to the COI forms do not work.

** For papers where more than one trial was reported, we summed the participant details across the studies and report the average values.
