# Supplementary material for: Characterisation of trials where marketing purposes have been influential in study design: a descriptive study
Source: Trials. 2016 Jan 21;17:31. doi: 10.1186/s13063-015-1107-1 (PMC4720997; doi:10.1186/s13063-015-1107-1)
Supplement: Additional file 2: — Letter to editors at NEJM, JAMA, Lancet and Annals of Internal Medicine. (DOCX 12 kb) [file 13063_2015_1107_MOESM2_ESM.docx]

Dear Dr

We recently presented the preliminary findings of a study we are doing at the Peer Review Congress which some of your colleagues attended and may have mentioned to you (see attached abstract). 

The background to this study is that we are concerned that an increasing number of trials are getting published that appear to be more about marketing than science or where marketing interests have undermined the scientific quality.  The purpose of our study is to try to characterise these trials with a view to identifying features that editors, reviewers and readers should be aware of when assessing trials.  However, characterising these trials using quantitative analysis is proving to be difficult.  Typical characteristics of marketing-driven trials appear to include the recruitment of small numbers of patients from numerous study sites for a common disease; misleading abstracts that do not report clinically relevant findings, primary outcomes of limited or unproven clinical relevance, and conclusions that focus on secondary end-points and surrogate markers, sometimes even failing to mention non-attainment of primary outcomes.

We analysed all trials published in the top six general medical journals in 2011 and six members of our study team reached a consensus that 41/207 (20%) appear to be more focused on marketing than answering a valid clinical question.  We plan to make our dataset available when we publish the study. However, in the meantime, the editor of JAMA has suggested that editors are given the opportunity to give feedback on the trials appearing in their own journals.  He intends to give us feedback over the JAMA trials.  We thought that you might also want to do this. 

I have attached a list of trials published in your journal on which our team reached a consensus that they appear to be primarily marketing driven.  We are interested to know whether you considered that they might partly be marketing trials when they were being reviewed, and how your editors approach the possibility of marketing as a driver in trials? To aid our understanding and inform our analysis we would also like copies of the anonymised peer review comments relating to these articles, if you were willing to share these with us in complete confidence.

We feel that further information from your editors about these trials might help us better characterise these studies and the degree to which they are influenced by marketing interests. If you would like any more information about our study, please do not hesitate to contact me.


Yours sincerely

Sara Schroter

On behalf of Carl Heneghan, Richard Lehman, Joe Ross, Druin Burch, Rafael Perera, Fiona Godlee and Ginny Barbour
